# Host–pathogen interactions in bacterial meningitis

**DOI:** 10.1007/s00401-015-1531-z

**Published:** 2016-01-07

**Authors:** Kelly S. Doran, Marcus Fulde, Nina Gratz, Brandon J. Kim, Roland Nau, Nemani Prasadarao, Alexandra Schubert-Unkmeir, Elaine I. Tuomanen, Peter Valentin-Weigand

**Affiliations:** Department of Biology and Center for Microbial Sciences, San Diego State University, San Diego, CA USA; Department of Pediatrics, University of California San Diego School of Medicine, La Jolla, CA USA; Institute for Microbiology, University of Veterinary Medicine, Bischofsholer Damm 15, 30173 Hannover, Germany; Centre for Infection Medicine, Institute of Microbiology and Epizootics, Freie Universität Berlin, Berlin, Germany; Department of Infectious Diseases, St Jude Children’s Research Hospital, Memphis, TN USA; Department of Geriatrics, Evangelisches Krankenhaus Goettingen-Weende, Goettingen, Germany; Institute for Neuropathology, University Medicine Goettingen, Goettingen, Germany; Division of Infectious Diseases, Children’s Hospital Los Angeles, University of Southern California, Los Angeles, CA USA; Institute of Hygiene and Microbiology, University of Wuerzburg, Würzburg, Germany

**Keywords:** Neuroinfectiology, Bacterial meningitis, Pneumococci, Meningococci, Group B Streptococcus, *Streptococcus suis*, *Escherichia coli* K1

## Abstract

Bacterial meningitis is a devastating disease occurring worldwide with up to half of the survivors left with permanent neurological sequelae. Due to intrinsic properties of the meningeal pathogens and the host responses they induce, infection can cause relatively specific lesions and clinical syndromes that result from interference with the function of the affected nervous system tissue. Pathogenesis is based on complex host–pathogen interactions, some of which are specific for certain bacteria, whereas others are shared among different pathogens. In this review, we summarize the recent progress made in understanding the molecular and cellular events involved in these interactions. We focus on selected major pathogens, *Streptococcus pneumonia*, *S. agalactiae* (Group B Streptococcus), *Neisseria meningitidis*, and *Escherichia coli* K1, and also include a neglected zoonotic pathogen, *Streptococcus suis*. These neuroinvasive pathogens represent common themes of host–pathogen interactions, such as colonization and invasion of mucosal barriers, survival in the blood stream, entry into the central nervous system by translocation of the blood–brain and blood–cerebrospinal fluid barrier, and induction of meningeal inflammation, affecting pia mater, the arachnoid and subarachnoid spaces.

## Introduction

**Fig. 1:**
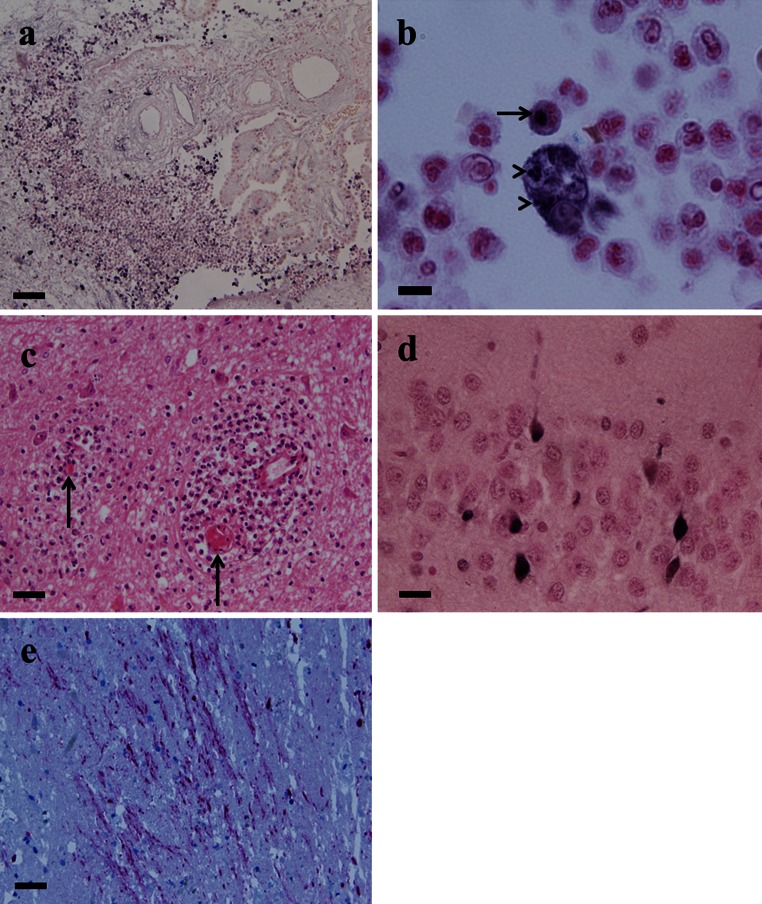
Inflammation and neuronal injury in human bacterial meningitis. **a** Strong infiltration of the right lateral ventricle by granulocytes and monocytes in *Neisseria meningitidis* meningitis. The double-strand DNA breaks in the nuclei of apoptotic granulocytes are stained *black* (in situ tailing counterstained with nuclear fast red, ×10). **b** Macrophage after phagocytosis of apoptotic granulocytes (*black, arrowheads*) and granulocyte at the beginning of the apoptotic process indicated by partial staining of its nucleus (*arrow*) (*N. meningitidis* meningitis, in situ tailing counterstained with nuclear fast red, ×100). **c** Thrombosis of two small vessels (*arrows*) and strong perivascular mainly granulocytic infiltrates in the thalamus, *Streptococcus pneumoniae* meningitis (haematoxylin–eosin, ×20). **d** Apoptosis of granule cells in the dentate gyrus of the hippocampal formation, otogenic bacterial meningitis (in situ tailing counterstained with nuclear fast red, ×40). **e** Diffuse axonal injury, *S. pneumoniae* meningitis (amyloid precursor protein immunohistochemistry, counterstaining with hemalum, ×20). *Bars* represent 120 μm (**a)**, 12 μm (**b)**, 60 μm (**c)**, 30 μm (**d**), 60 μm (**e**)

Bacterial meningitis is a serious threat to global health. *Neisseria meningitidis*, *Streptococcus pneumoniae* and *Haemophilus influenzae* type b are most commonly associated with bacterial meningitis in infants and adults [150]. In sub-Saharan Africa, also called the ‘meningitis belt’, *N. meningitidis* is a leading cause of large epidemics of meningococcal meningitis. Further bacteria that cause meningitis in children and adults include Group B Streptococcus (GBS), *Escherichia coli* K1, non-typhoideal *Salmonella*, *Klebsiella* spp., *Staphylococcus aureus*, *Listeria monocytogenes*, *Mycobacterium tuberculosis* and the neglected porcine zoonotic pathogen *Streptococcus suis*. Many of the meningeal pathogens are able to colonize the skin and different mucosal surfaces of healthy individuals. In certain cases, bacteria penetrate host cellular barriers to initiate a local infection that can result in systemic spread. An association between high-level bacteremia and development of meningitis has been suggested for some bacteria [83, 108]. This implies that survival in the blood is an important virulence trait of meningeal pathogens. Following bloodstream survival or by spread from infectious foci in the vicinity of the brain (mastoiditis, sinusitis), bacteria will ultimately invade the central nervous system (CNS), resulting in inflammation of the meninges, increased blood–brain barrier (BBB) permeability, cerebrospinal fluid (CSF) pleocytosis, and infiltration of the nervous tissue (Fig. 1). Subsequent CNS tissue injury (Fig. 1) results from apoptotic neuronal injury, cerebral ischemia, edema, hydrocephalus and increased intracranial pressure [96] and is caused by both toxic bacterial products and host inflammatory pathways initiated to clear the infection. In particular, the excessive inflammatory response of neutrophils (PMNs) has been associated with increased CNS injury [57] (Fig. 1). This review summarizes recent progress made in our understanding of host–pathogen interactions in bacterial meningitis, exemplified by four of the most common pathogens, *S. pneumoniae*, Meningococcus, GBS, and *E. coli* K1, and a rare but neglected pathogen, *S. suis*).

## Common steps and mechanisms in pathogenesis of bacterial meningitis

Pathogens causing meningitis often colonize mucosal surfaces and show similar patterns of disease progression. Thus, it is plausible that they share common strategies to advance from the mucosa into the blood stream and further into the brain. An overview of main similarities and differences of the pathogens described in following chapters is given in Table 1. Many bacteria bind to extracellular matrix proteins, e.g., laminin, collagen or fibronectin, to facilitate initial attachment preceding invasion. In addition, some bacterial adhesins, e.g., of *N. meningitidis*, also bind to members of the CEACAM family of cell adhesion molecules, others, e.g., OmpA of *E. coli* K1, recognize specific glycoproteins in a lectin-like fashion. Binding of bacterial adhesins to specific host cell receptors may lead to a signal transduction resulting in tight bacterial attachment to or internalization by the host cells. As outlined above (see “*S. pneumoniae* meningitis”) “innate invasion” is a common entry mechanism that counteracts innate immune mechanisms and employs molecular mimicry, as exemplified by PCho mimicking the chemokine PAF. A hallmark of many bacteria infecting the CNS is their ability to survive in the blood stream by either avoiding or protecting against phagocytosis, e.g., by expression of a capsule (*S. suis*) or by entering and persisting in PMNs or macrophages (*E. coli* K1). However, sustained bacteremia is not always a prerequisite for bacterial entrance to the CNS, since meningitis can also be caused by direct invasion from neighboring infected tissues. Nevertheless, all bacteria have to breach certain barriers, such as the BBB and blood–CSF barrier (B-CSFB), to get access to the brain. Translocation across such barriers may occur via a para- or transcellular process, depending on the virulence traits expressed by the pathogen. Cytolytic toxins, e.g., those expressed by *S. pneumoniae*, GBS, *S. suis* and *E. coli*, can damage host cells thereby leading to disruption of the barrier and mediation of paracellular invasion. Transcellular breaching of barriers is based on intracellular invasion, which often involves bacterial exploitation or “hijacking” of signal platforms and pathways, as exemplified by *N. meningitidis*. Once the pathogen has reached the brain, bacteria (or bacterial components) are recognized by resident immune cells, such as microglia and astrocytes, leading to their activation. Furthermore, circulating professional immune cells, such as granulocytes and monocytes/ macrophages, are attracted and subsequently infiltrate the infected brain parenchyma (Fig. 1). Especially in the neonate host, the resulting antibacterial immune response might be overwhelming and not well orchestrated, leading to a pronounced neuronal damage and even death. If the host survives infection, pathogen-specific post-infectious sequelae, such as deafness, blindness or certain kinds of retardation might be the result.

**Table 1:** Main similarities and differences of bacterial pathogens causing meningitis

	*Streptococcus pneumoniae*	*Neisseria meningitidis*	Group B *Streptococcus*	*Streptococcus suis* ^a^	*Escherichia coli* K1
Nature of the pathogen	Gram-positive cocci, encapsulated, serotype diversity, extracellular	Gram-negative cocci, encapsulated, serogroup diversity, clonal complexes, extracellular	Gram-positive cocci, encapsulated, serotype diverse, Type III most common, extracellular	Gram-positive cocci, encapsulated, serotype diversity, extracellular	Gram-negative rod shaped, K1 capsular polysaccharide
Affected age group	Children <5 years Adults >50 years	Children <5 years	<3 months	Adults	<3 months
Site(s) of entry and colonization	Nasopharynx, Lung	Nasopharynx	Hematogenous spread from mother to infant, nasopharynx, intestinal tract	Nasopharynx, cutaneous wounds, intestinal tract	Hematogenous spread from mother to infant, nasopharynx, intestinal tract
Factors involved in bacterial adherence and invasion	Cell wall-anchored proteins, cytolysin, capsule	Capsule, type IV pili, outer membrane proteins (Opa, Opc, FBA, ACP, MspA)	Cell wall-anchored proteins, hemolysin, capsule, LTA, pili	Cell wall-anchored proteins, cytolysin, capsule, LTA	OmpA, K1 capsule, CNF1, Fimbriae, IbeA
Mechanisms of survival and dissemination in the blood	Capsule-dependent protection, complement inhibitors	Capsule-dependent protection, complement inhibitors	Capsule-dependent protection, complement inhibitors, intracellular survival	Capsule-dependent protection, complement inhibitors, monocytes as “Trojan Horse”	OmpA- and capsule-dependent protection, survival in PMNs and macrophages
Mode(s) of entry into the CNS	Invasion across the BBB and B-CSFB	Invasion across the B-CSFB	Invasion across the BBB and B-CSFB?	Invasion across the BBB and the B-CSFB	Invasion across the BBB
Causes of tissue damage in the CNS (cerebral ischemia, edema, hydrocephalus, increased intracranial pressure)	Cytotoxin, cell wall-TLR2 induced inflammation, neuronal apoptosis, increased BBB permeability	Release of inflammatory mediators, increased BBB permeability, neuronal apoptosis, LPS	Hemolysin induced inflammation, tight junction disruption, increased BBB permeability	Release of inflammatory mediators, increased BBB permeability, neuronal apoptosis? Cytotoxins?	Inflammation, neuronal apoptosis, increased BBB permeability, CNF1?
Pathology and clinical symptoms	Meningitis, sepsis, pneumonia	Meningitis, sepsis	Meningitis, sepsis, pneumonia	Meningitis, endocarditis, peritonitis, pneumonia, arthritis, sepsis, STSLS	Sepsis, meningitis
Possible sequelae	Deafness, learning deficits, paralysis	Deafness, neuro-developmental deficits	Learning deficits, deafness, cortical blindness, seizures	Deafness	Learning deficits, deafness, cortical blindness

*BBB* blood–brain barrier, *B-CSFB* blood–cerebrospinal fluid barrier, *STSLS* streptococcal septic shock-like syndrome, *LTA* lipoteichoic acid

^a^ *S. suis* can cause meningitis in pigs and humans. This table only shows features of human infections

## *Streptococcus pneumoniae* meningitis

*Streptococcus pneumoniae*, a Gram-positive extracellular pathogen, is one of the most common etiologic agents of bacterial meningitis worldwide affecting predominantly young children and the elderly. While more commonly a quiescent colonizer of the nasopharynx, this bacterium causes mild infections such as otitis media and sinusitis but also life-threatening conditions such as pneumonia, bacteremia and meningitis. Pneumococcal meningitis is characterized by a high mortality rate (20–30 %) due to complications such as brain edema, cerebral ischemia and increased intracranial pressure arising by an excessive immune response as well as damage by the pathogen itself. Survivors suffer from long-term neurological deficits such as hearing loss and cognitive impairment. Recently discovered reasons for long-term neurological sequelae in pneumococcal meningitis may be focal or diffuse axonal injury (Fig. 1) [87] and synapto- and dendritotoxicity mediated by pneumolysin and glutamate [155].

Most of the findings regarding the pathophysiology of pneumococcal meningitis are either derived from brain autopsies (representing only fatal cases) or from animal models that aim to closely mimic clinical features of human disease. The most prominent models are the mouse, the rabbit and the rat. The use of knockout technology made the mouse a useful model to study the host response to the pneumococcus during meningitis [94]. Also, hippocampal neuronal apoptosis [78] and cortical brain damage have been observed [55] with this model. The rabbit was used to study meningitis-related processes within the CSF, e.g., bacterial growth, antibiotic penetration and components of the immune response [24, 92]. In the rabbit model, apoptotic damage occurs in the dentate gyrus of the hippocampal formation [14, 161]. This form of neuronal injury is present in approx. 70 % of human autopsy cases [88] (Fig. 1d). In the infant rat model, cortical and hippocampal damage have been observed that closely resembles the pattern of necrotic and apoptotic neuronal injury in human pneumococcal meningitis [66, 67]. Results in the adult rat are less consistent, since some studies see significant damage in the cortex [13, 135] whereas others not [56]. This might be due to differences in the pneumococcal strain used for the study and also the parameters that have been chosen as a readout.

### Bacterial invasion and dissemination

To cause infection of the CNS, the pneumococcus has to enter the respiratory tract, escape mucous defenses and either translocate into the bloodstream to cause invasive pneumococcal disease (IPD) or cause mastoiditis or sinusitis and spread locally through skull defects or along vessels penetrating the skull. To enter the bloodstream, an armory of virulence factors is used including surface proteins, polysaccharide capsule and cell wall. Interestingly, the two other major meningeal pathogens of children, the meningococcus and *Haemophilus influenzae,* share the same pattern of disease progression, which led to the hypothesis that these pathogens use a common strategy to advance from the respiratory mucosa into the bloodstream and further into the brain. This common entry mechanism, called “innate invasion”, counteracts innate immune mechanisms and employs molecular mimicry to promote invasion.

Innate invasion is initiated by the binding of the bacteria to the respiratory epithelium. The adhesin, choline-binding protein A (CbpA), binds to the polymeric immunoglobin receptor (pIgR) thereby initiating bacterial translocation across the nasopharyngeal epithelium [159]. High titer bacteremia then promotes the development of meningitis by bacterial host interactions at the BBB. At the cerebrovascular endothelium, CbpA binds laminin receptor (LR) [91]. Importantly, *Neisseria meningitidis* and *H. influenzae* use a CbpA homolog to bind LR for attachment to the BBB [91]. This observation led to the development of a CbpA-based-vaccine that crossprotects against these pathogens [75]. In addition to LR, platelet endothelial cell adhesion molecule-1 (PECAM-1, also known as CD31) and the lectin-like domain of the pneumococcal neuraminidase A (NanA) have been shown to contribute to pneumococcal attachment to BBB endothelial cells [47, 142].

### Bacterial translocation into the CNS

After bacterial attachment to epithelial or endothelial cells, translocation across the barriers is again mediated by the innate invasion process. Phosphorylcholine (PCho) is displayed on the surface of virtually all respiratory pathogens and, by mimicking the chemokine PAF, mediates binding to the human platelet activating factor receptor (PAFr) [21]. In the case of the pneumococcus, PCho is added to cell wall teichoic acid and lipoteichoic acid in a phase variable manner [22]. Binding of PCho to the PAFr leads to clathrin-mediated uptake of bacteria into a vacuole, thereby facilitating intracellular bacterial translocation from the bloodstream into the brain [103]. Experiments using PAFr antagonists or PAFr-deficient mice revealed that bacteria fail to invade the bloodstream or CNS when this receptor is not available [36, 107]. The interaction of PCho with PAFr is counteracted by the host innate immunity components C-reactive protein (CRP) and surfactant, both of which target PCho [43]. The pneumococcus has also been described to use the vitronectin-αvβ3 integrin complex for invasion of epithelial and endothelial cells [9].

In addition to receptor-mediated uptake into host cells, the pneumococcus gains access into the CNS paracellularly by disruption of BBB integrity. This process is mediated by the cholesterol-dependent cytolysin pneumolysin [162] and the α-glycerophosphate oxidase GlpO [71] that creates H_2_O_2_ thereby causing apoptosis of brain microvascular endothelial cells. Hyaluronidase might also contribute to meningitis by degradation of components of the extracellular matrix [59]. Further, a secreted version of NanA appears to modulate tight junction protein expression by activation of TGF-β resulting in an increase of BBB permeability (unpublished results). But sustained bacteremia is not always a prerequisite for the pneumococcus to enter the CNS. In adults, meningitis can be caused by direct invasion from neighboring infected tissues. A recent study revealed that pneumococcal carriage in the nasopharynx can lead to pneumococcal invasion of the brain via retrograde axonal transport along olfactory neurons [146].

### Immune activation and inflammatory response in the brain

Once the pneumococcus gains access to the CNS, it takes advantage of the limited host defense mechanisms in this compartment and rapidly multiplies within the cerebrospinal fluid (CSF). During multiplication, bacteria release components that are highly immunogenic and are recognized by pattern recognition receptors (PRRs) on the surface of antigen-presenting cells that are present in low numbers in the CSF. Immune recognition of these bacterial components results in a strong inflammatory response leading to BBB impairment due to recruitment of leukocytes (Fig. 1a), vascular deregulation, vasculitis and occlusion of vessels (Fig. 1c) which cause increased intracranial pressure. Interestingly, inflammation within the CNS is detectable at high titer bacteremia even prior to when bacteria cross the BBB [48].

The entire symptom complex of meningitis can be triggered in the absence of live bacteria, when only components of the bacterial cell wall are intracisternally inoculated into animals [141]. This observation is especially important in the clinical setting since bacterial lysis caused by antibiotic treatment leads to explosive cell wall release, resulting in an increased host response and disease severity [86]. The most important PRRs responsible for the detection of the pneumococcus in the CNS are members of the Toll-like receptor (TLR) family (TLR2, TLR4 and TLR9) and NOD2 that belongs to the family of NOD-like receptors (NLRs) [82]. TLR2 recognizes pneumococcal cell wall, lipoproteins as well as lipoteichoic acid, whereas TLR4 detects pneumolysin and TLR9 senses bacterial DNA that is released during autolysis [58]. In addition, muramyl peptides from pneumococcal peptidoglycan are recognized by intracellular NOD2 [69] and PCho-bearing teichoic acids bind to the PAFr [21]. Inflammasome-mediated recognition of the pneumococcus also contributes to the host innate immune response. The inflammasome component NALP3 has been shown to be a critical player in this process [45].

Engagement of the inflammatory response activates various signaling cascades resulting in the production of pro-inflammatory mediators that orchestrate an efficient immune response. Patients with pneumococcal meningitis show high levels of pro-inflammatory cytokines such as TNF-α, IL-1β, IFN-γ, IL-2, IL-6 and IL-12, anti-inflammatory cytokines (IL-10 and TGF-β) and chemokines such as CXCL8 (IL-8) CCL3 (MIP-1a) and CCL2 (MCP-1) in their CSF [19]. The secreted chemokines act together with other chemoattractants (e.g., PAF, reactive oxygen and nitrogen species) and the complement system to attract highly activated PMNs to the brain. These cells cross the BBB through the tight junctions of the endothelial cells that form this barrier in a multistep process involving integrins and selectins, leading to CSF pleocytosis [82]. Matrix metalloproteases (MMPs) produced by neutrophils, neurons, glia cells and endothelial cells upon infection have been shown to play an important role in this process by lysing the subendothelial basement membrane thereby promoting BBB breakdown and leukocyte invasion [67]. However, the invading leukocytes present in the CSF do not efficiently phagocytose the pneumococcus. This might partly be due to the lack of sufficient concentrations of complement components and immunoglobulin to opsonize the pathogen.

Activation of the immune response and the rapid influx of leukocytes into the brain also come at a cost for the host. Activated immune cells within the brain, such as microglia, astrocytes and infiltrating leukocytes as well as microvascular endothelial cells, amplify the cascade of pro-inflammatory cytokines and cytotoxic agents that cause tissue damage in cortical and subcortical structures [82]. Inhibition of many steps in the inflammatory cascade, such as neutrophil recruitment, improves the clinical outcome of meningitis by decreasing neuronal loss [5]. Therefore, antibiotic treatment of community-acquired meningitis is most often accompanied by administration of dexamethasone, to protect the brain from the abrupt increase of inflammation during early bacterial death.

## Meningococcal meningitis

*Neisseria meningitidis* (meningococci) is a frequently found asymptomatic colonizer of the upper respiratory tract, which under certain circumstances may penetrate the mucosal membrane, reach the bloodstream and cause severe septicemia and/or meningitis. The interaction of *N. meningitidis* with human endothelial cells lining the blood vessels of the blood–CSF barrier (B-CSFB) is a prerequisite for the development of meningitis. Over the past decade, important advances have been made in understanding the molecular mechanisms of the interaction of *N. meningitidis* with endothelial cells of the B-CSFB. The following chapter will highlight the current knowledge about the specific adhesion-receptor interactions that allow *N. meningitidis* to tightly bind to the targeted host cell with a focus on the induced signaling pathways.

### Bacterial invasion and dissemination

Bacterial binding to brain endothelial cells is a prerequisite for successful penetration into the CSF. Large colonies of *N. meningitidis* have been found on the capillaries of the subarachnoideal space, in the parenchyma and in the choroid plexus in histological sections of brain tissues of postmortem samples [100]. To establish binding to host cells, *N. meningitidis* possess a variety of determinants that contribute to these interactions including type IV pili, outer membrane proteins (Opa and Opc), and a number of newly described so-called minor adhesion or adhesion-like proteins, such as the adhesin complex protein (ACP) or the autotransporter meningococcal serine protease A (MspA) (for a review see [148]).

Type IV pili (Tfp) are polymeric filaments that are found in a variety of Gram-negative bacteria. They mediate the initial contact of *N. meningitidis* to eukaryotic cell surfaces, and are involved in bacterial movement, also known as ‘twitching motility’, and transformation competence. Tfp in *Neisseria* spp. are composed of one main component, the major pilin, PilE, that assembles into a helical fiber. The helical assembly of pilin into fibers relies on proteins located in or in the vicinity of the cytoplasmatic membrane.

**Fig. 2:**
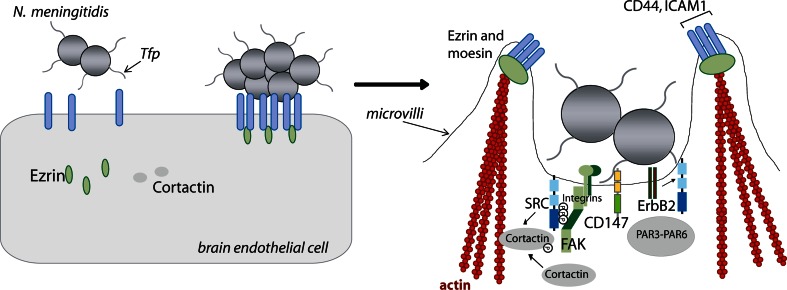
Schematic illustration of the initial steps of the interaction of *Neisseria meningitidis* with brain endothelial cells. **a** *N. meningitidis* adheres to brain microvascular endothelial cells via type IV pili. (**b**, Detailed) following initial bacterial adhesion, type IV pili (Tfp) mediate the recruitment and the activation of several transmembrane proteins, including ICAM-1 and CD44 as well as accumulation of ezrin and moesin, two members of the ezrin–radixin–moesin protein family. The formation of these so-called ‘cortical plaques’ induces the formation of microvilli-like protrusions that surround the bacteria, protect bacterial colonies from the blood flow shear stress and initiate their internalization within vacuoles. A result of the formation of ‘cortical plaques’ is the replacement of the polarity complex proteins PAR3/PAR6/αPKC that are usually localized at the intercellular junctions. Moreover, the meningococcal Opc protein confers a tight association of the bacterium to fibronectin and/or vitronectin mediating binding to endothelial integrins (*light and dark green ovals*). This interaction leads to activation of non-receptor tyrosine kinases (Proto-oncogene tyrosine-protein kinase c-Src and focal adhesion kinase (FAK) and receptor tyrosine kinases (ErbB2), resulting in phosphorylation and activation of cortactin and cytoskeletal rearrangement (actin monomers, *red* globules)

Considerable efforts have been undertaken to determine the binding receptor of Tfp on eukaryotic cells. CD46 or membrane co-factor protein has been described as a proposed host cell receptor for Tfp [51], but the role of CD46 as a host cell receptor has been controversial. In addition, the platelet activating factor (PAFr) was described as a pilus receptor targeted on airway epithelial cells [49]. Recent published data now shed new light on a possible pilus receptor targeted on brain endothelial cells. Bernard et al. [11] showed that *N. meningitidis* utilizes CD147, a member of the immunoglobulin superfamily, for Tfp-dependent adhesion to endothelial cells and demonstrated the central role of CD147 for vascular colonization of pathogenic meningococci. Tfp-mediated adhesion to CD147 was shown to involve both PilE and the minor pilin PilV. Interfering with Tfp/CD147 interaction blocked binding of meningococci to human endothelial cells in vitro and importantly also prevented colonization of vessels in human brain tissue explants ex vivo [11]. Furthermore, PilE- and PilV-dependent colonization of *N. meningitidis* to endothelial vessels was verified in vivo using a model of severe combined immunodeficiency mice grafted with human skin [11]. Interestingly, both pilins have also been reported to activate the G protein-coupled β_2_-adrenergic receptor (β_2_-AR) that serves primarily as a signaling receptor [17]. In response to bacterial adhesion and the formation of meningococcal microcolonies, β_2_-AR is recruited to the apical surface of the endothelial cell underneath the microcolonies [17]. The interaction of PilE and PilV with the extracellular N-terminal domain of β_2_-AR most likely modifies the conformation of the receptor resulting in the activation of β-arrestin-mediated signaling pathways [17]. However, *N. meningitidis*-induced activation of β_2_-AR does not elicit G protein-mediated signal transduction. The receptor activation by meningococci is biased toward the β-arrestin pathway. Trapped β-arrestin recruits ezrin and the non-receptor tyrosine kinase (RTK) c-Src, which phosphorylate cortactin (Fig. 2). Secondly, β-arrestin leads to the accumulation of β-arrestin-interacting proteins, such as VE-cadherin and p120-catenin, into so-called ‘cortical plaques’ underneath bacterial microcolonies. This accumulation was shown to result in depletion of intercellular junctions, a mechanism described in more detail below.

The outer membrane proteins comprise the colony opacity-associated (Opa) proteins and Opc. Though outer membrane proteins are partially masked by the polysaccharide capsule, they also efficiently support adhesion and invasion to eukaryotic cells especially on cells of high receptor density as would be induced in inflammatory conditions and/or lateral receptor aggregation [12]. Most Opa proteins have been demonstrated to bind to members of the human carcinoembryonic antigen-related cell adhesion molecule (CEACAM) family on epithelial cells (for reviews see [111]). In addition, some Opa proteins can bind to heparan sulfate proteoglycans (HSPG) or to integrins via the extracellular matrix proteins vitronectin and fibronectin or saccharides [147]. Although binding of the Opa proteins to CEACAM receptors has been described in detailed for epithelial cells, there is only limited information about the role of CEACAMs on brain endothelial cells and the contribution of the Opa/CEACAM receptor interaction during meningococcal adhesion and/or invasion into brain endothelial vessel cells.

The outer membrane protein Opc is particularly implicated in host cell invasion of endothelial cells, including brain endothelial cells [148]. Opc is a beta barrel protein with five surface loops encoded by a single gene (*opcA*) and is antigenically stable. The level of Opc protein expression is phase variable, due to the transcriptional regulation of a homopolymeric polycytidine (Poly-C) stretch, within the promoter region [112]. The number of nucleotide repeats determines the promotor strength and binding efficacy of the RNA polymerase. Opc is expressed by several virulent *N. meningitidis* lineages, but is absent from certain epidemic clones (ET-37/ST-11 clonal complex) and a few random endemic isolates [112]. Interestingly, two epidemiological studies reported outbreaks where meningococcal strains of the ST-11 complex tend to cause severe sepsis with fatal outcome, but rarely meningitis [153], suggesting Opc as a major candidate that enhances the bacterial ability to cause meningitis.

The Opc protein can bind directly to components of the extracellular matrix (ECM) and serum proteins, such as vitronectin or fibronectin [110, 143]. In addition, Opc may indirectly bind to fibronectin and vitronectin via heparin, since both fibronectin and vitronectin are heparin-binding proteins. By binding to fibronectin or vitronectin bacterial adhesins can also target proteoglycans. The tight association of Opc to vitronectin and/or fibronectin in turn mediates binding of meningococci to their cognate receptor, endothelial αVβ3 integrin (vitronectin receptor) [110] and/or α5β1-integrin (fibronectin receptor) [143] on brain vessel cells.

Besides the activation of the non-RTK c-Src in a Tfp-dependent manner, meningococcal binding to integrins via Opc also leads to activation of c-Src. Detailed analysis revealed that pharmacological inhibition of c-Src activity as well as genetic interference with c-Src expression interfered with bacterial uptake [125]. The role of this kinase in bacterial uptake was further verified in Src-deficient fibroblasts that are impaired in their ability to internalize *N. meningitidis*. Similar to the role of c-Src, pharmacological inhibition and genetic ablation of the focal adhesion kinase (FAK) also blocked bacterial uptake [124]. As a downstream target cortactin is phosphorylated downstream of integrin-Src activation, demonstrating that a cooperative interplay between FAK, Src and cortactin occurs during meningococcal uptake by brain endothelial cells (Fig. 2) [124].

Beside activation of non-RTKs *N. meningitidis* can activate RTKs and thus modulate host cell signaling pathways for their purposes. A phosphorarray screen demonstrated activation of further interesting RTKs and key signaling nodes [126]; however, their functional significance in the context of *N. meningitidis* interaction with brain endothelial cells remains to be determined. Interestingly, the signaling mechanisms which are involved in bacterial entry into brain endothelial cells may differ from those that are involved in the release of cytokines and chemokines: this is evidenced for example for the *N. meningitidis* infection of the cell line HBMEC, which requires c-Jun kinases 1 and 2 (JNK1 and JNK2) activation for bacterial uptake, but not for cytokine release. Cytokine release instead, such as IL-6 and IL-8 from infected HBMEC involves the p38 mitogen-activated protein kinase (MAPK) pathway [128].

### Bacterial translocation into the CNS

The tight interactions of the bacterial adhesins/invasins with their respective receptors on brain endothelial cells and subsequent induced uptake favor the strategy for a transcellular pathway for meningococcal transversal across the tight B-CSFB. A paracellular pathway would require opening of the tight junctions or even breakdown of the barrier as a consequence of induced apoptosis or cytotoxicity. The latter is unlikely, since subarachnoid hemorrhage is a rare complication of bacterial meningitis. Recent publications have highlighted mechanisms that facilitate a paracellular route for *N. meningitidis* translocation into the CNS [18, 114]. When adhering to endothelial cells, *N. meningitidis* induces local elongation of the cell resembling epithelial microvilli structures [33]. These microvilli-like structures surround the bacteria and initiate their internalization within vacuoles [33]. They increase the cell membrane surface to facilitate bacterial adhesion and contribute to resistance against shear stresses in the bloodstream [72]. Interestingly, formation of these cellular protrusions was also observed ex vivo in histological section of a choroid plexus capillary from a postmortem sample [85]. These protrusions are enriched in ezrin and moesin, two members of the ezrin–radixin–moesin (ERM) protein family, and several transmembrane proteins, including ICAM-1, ICAM-2 and CD44 [33]. Recruited integral membrane proteins, adapter proteins and the actin cytoskeleton form specific molecular complexes also referred to as ‘cortical plaques’. Interestingly, as a result of the formation of ‘cortical plaques’ replacement of proteins usually localized at the intercellular junctions occurs. In particular, the polarity complex PAR3/PAR6/αPKC proteins are recruited at the meningococcal adhesion site [18] with depletion at the cell–cell interface and opening of the intercellular junctions of the brain–endothelial interface. The formation of the mislocated adherence junctions may open up a paracellular route for *N. meningitidis* transversal into the CNS [18]. Further altering of cellular junctional proteins in vitro has been shown for the tight junction protein occludin using the HBMEC cell line as an in vitro model [114]. Prolonged time of infection resulted in proteolytic cleavage of occludin by the matrix-metalloproteinase MMP-8 [114]. As a consequence of proteolytic cleavage occludin disappears from the cell periphery and is cleaved to a smaller sized 50-kDa protein in infected cells resulting in endothelial cell detachment and increased paracellular permeability [114].

Bacterial binding and subsequent uptake by the host cells not only implicates binding to specific ligand receptor, but requires a re-organization of receptor molecules and of signaling molecules in the cell membrane. Recent studies indicate that specialized domains of the cell membrane, termed rafts, are central for the spatial organization of receptors and signaling molecules. Bacteria can hijack and take advantage of these signaling platforms activated within specialized membrane domains.

Studies in the last years revealed that lipids in the cell membrane are not randomly distributed but seem to be organized. Sphingomyelin is the most prevalent sphingolipid and predominantly localizes in the anti-cytoplasmatic leaflet of cell membranes and intracellular vesicles. It is composed of a highly hydrophobic ceramide moiety and a hydrophilic phosphorylcholine headgroup. Hydrolysis of sphingomyelin results in the release of ceramide which alters the biophysical properties of membranes. Ceramide molecules spontaneously interact with each other to form ceramide-enriched domains and, due to their biophysical properties, ceramide-enriched membrane domains then fuse into extended platforms which span a few hundred nanometers to several micrometers. In addition to altering membrane fluidity and rigidity, ceramide-enriched platforms serve to sort and eventually concentrate membrane receptors and membrane proximal signaling components thereby amplifying cellular responses and signal transduction. Ceramide-enriched platforms have been implicated in the internalization of different bacteria [44]. Recent published data now revealed that *N. meningitidis* is also capable to activate the acid sphingomyelinase (ASM) in brain microvessels thus leading to generation of ceramide and the formation of ceramide-enriched platforms [123]. Mechanistically, ASM activation relies on binding of *N. meningitidis* to its attachment receptor, HSPG, followed by activation of the phosphatidylcholine-specific phospholipase C. In addition, *N. meningitidis* infection promoted receptor (ErbB2) recruitment in ceramide-enriched platforms. Interestingly, meningococcal isolates of the ST-11 clonal complex, which rarely cause meningitis (see above), barely induced ASM and ceramide release correlating with significant lower bacterial uptake by brain endothelial cells [123]. These data indicate a differential activation of the ASM/ceramide system by the species *N. meningitidis* determining its invasiveness into brain endothelial cells.

### Immune activation and inflammatory response in the brain

Cytokine activation is an important event in the pathogenesis of meningococcal disease [149]. The acute inflammatory response is compartmentalized within the subarachnoid space and is characterized by the release of tumor necrosis factor α (TNF-α), IL-1β, IL-6, IL-8, MCP-1, MIP-α and G-CSF [149]. Interestingly, based on experiments with meningioma cells, *N. meningitidis* induce higher levels of the cytokines than the same number of *S. pneumoniae*, *H. influenza*e or *E. coli* K1 [46]. LPS is the major inflammatory modulin produced by *N. meningitidis,* however, several studies have shown that non-LPS components also contribute to cytokine secretion. The release of cytokines results in alteration of the vasculature of the meninges and in upregulation of different adhesion molecules on the endothelial cells, including selectins, intercellular adhesion molecules (ICAMs) and the vascular endothelial adhesion molecules (VECAMs). Circulating leukocytes, primarily neutrophils, are attracted by IL-8 and can pass between the activated endothelial cells entering the subarachnoid space. In parallel, proteins (mainly albumin), immunoglobulins and complement factors leak into the CSF. TNF-α and IL-1β are produced at the very early stage and can be found in a bioactive form in half of the patients on admission. The release of IL-6, IL-8, MCP-1 and MIP-α continues for a longer time or are upregulated to higher levels and can be detected in the majority of the patients during hospital admission.

## Group B Streptococcus meningitis

Group B Streptococcus (GBS) is a Gram-positive encapsulated bacterium possessing an array of virulence factors that enable it to produce serious disease in susceptible hosts, in particular the human newborn [73]. Notably, GBS is the leading cause of meningitis in the neonatal period [73]. Although advances in intensive care management and antibiotic therapy have changed GBS meningitis from a uniformly fatal disease to a frequently curable one, the overall outcome remains unfavorable. Morbidity is high with 25–50 % of surviving infants suffering permanent neurological sequelae, including cerebral palsy, mental retardation, blindness, deafness, or seizures [32]. The pathogenesis of neonatal GBS infection begins with the asymptomatic colonization of the female genital tract. Approximately 20–30 % of healthy women are colonized with GBS on their vaginal or rectal mucosa, and 50–70 % of infants born to these women will themselves become colonized with the bacterium [3]. Of the 10 different GBS capsular serotypes described, five (Ia, Ib, II, III, and V) are typically more associated with disease and account for the majority of cases worldwide [31]. GBS has more recently also been classified by sequence type (ST) based on an allelic profile of seven different loci, with the majority of GBS human isolates being ST-1, ST-17, ST-19, or ST-23 [50]. Interestingly there is a disproportionate burden of serotype III, ST-17 strains associated with neonatal invasive disease and meningitis [136]. The type III, ST-17 GBS clone has been referred to as the hypervirulent strain and accounts for the majority of GBS meningitis cases [136]. In this section, we review the mechanisms by which GBS is able to gain access to, and penetrate the BBB as well as highlight the response of the BBB to GBS with particular emphasis on newly described mechanisms of GBS BBB penetration.

Neonatal GBS infections are traditionally classified as two forms: early-onset disease (EoD) and late-onset disease (LoD). Early-onset infections typically occur in the first week of life, presenting acutely with pneumonia and respiratory failure complicated by bloodstream infection, septicaemia and sometimes meningitis. In contrast, GBS LoD occurs in infants up to 7 months of age, with more indolent symptom progression related to bacteremia and a high incidence (~50 %) of meningitis [3]. The pathophysiology of GBS meningitis varies according to age of onset. In EoD, autopsy studies demonstrate little or no evidence of leptomeningeal inflammation, despite the presence of abundant bacteria, vascular thrombosis and parenchymal hemorrhage [102]. By contrast, infants with LoD usually have diffuse purulent arachnoiditis with prominent involvement of the base of the brain [10]. These histopathological differences reflect underdevelopment of the host immunological response in the immediate neonatal period, with a higher proportion of deaths resulting from overwhelming septicemia. Clinical and neuropathologic studies have documented the clear association between bacterial meningitis and brain edema formation, increased intracranial pressure, seizure activity, arterial and venous cerebral vascular insults, and other neurologic sequelae [113]. A recent study found that GBS meningitis can be complicated by severe cerebrovascular disease, including arterial ischemic stroke and cerebral sinovenous thrombosis, and that these complications may be underestimated [140].

To produce meningitis, blood-borne GBS must typically penetrate the BBB and/or the B-CSFB. Ultimate disruption of BBB integrity may be due to the combined effect of bacterial entry and penetration of brain microvascular endothelial cells (BMEC), direct cellular injury by bacterial cytotoxins, and/or activation of host inflammatory pathways that compromise barrier function. It is apparent that the host immune response is incapable of controlling infection within the CNS and that this host inflammatory response may be responsible for many adverse events during bacterial meningitis. A very complex and integrated series of events involving host cytokines, chemokines, proteolytic enzymes, and oxidants appears to be responsible for meningitis-induced brain dysfunction. The development of GBS meningitis progresses through phases including (1) bloodstream survival and the development of bacteremia, (2) direct GBS invasion and disruption of the BBB, and (3) GBS multiplication in the CSF-containing subarachnoid and ventricular spaces, which induces inflammation with associated pathophysiologic alterations leading to the development of neural damage.

### Bacterial invasion and dissemination

An association between sustained high-level bacteremia and development of GBS meningitis has been suggested in humans and in experimental models of hematogeneous meningitis [73]. This observation implies that GBS bloodstream survival is an important virulence trait to avoid immune clearance by host immune cells, prior to CNS penetration. Neonates are particularly prone to invasive disease because of their quantitative or qualitative deficiencies in phagocytic cell function, specific antibody, or the classic and alternative complement pathways. In addition to these newborn host susceptibilities, GBS possess a number of virulence determinants that promote bloodstream survival by thwarting key components of effective opsonophagocytic killing by host leukocytes [73]. The sialylated GBS capsular polysaccharide (CPS) represents one of the most critical factors for limiting the effectiveness of host complement and phagocytic defense. While serotype III GBS strains have accounted for a majority of LoD and meningitis [3, 136], all serotypes contain a terminal-linked sialic acid bound to galactose in an α2 → 3 linkage [73]. Bacterial surface sialylation may have evolved to mimic host ‘self’ antigens, allowing GBS to avoid immune detection, manipulate phagocyte function and dampen the immune response to GBS infection. The sialic acid moiety provides anti-phagocytic protection by impairing deposition of opsonically active complement C3 on the bacterial surface, but also activates anti-inflammatory receptors on host leukocytes promoting GBS persistence in the blood stream [73]. Isogenic GBS mutants lacking CPS or capsular sialylation are more susceptible to neutrophil killing and are less virulent in rodent and zebrafish infection models [93, 109].

Once GBS is engulfed by phagocytic cells, the bacterium may be able to resist toxic reactive oxygen species (ROS) produced in the phagolysosome to survive intracellularly. GBS produces an orange carotenoid pigment, a property unique to GBS among hemolytic streptococci, associated with the *cyl* operon encoding the β-hemolysin/cytolysin cytotoxin [73]. The free-radical scavenging properties of this associated carotenoid neutralize hydrogen peroxide, superoxide, hypochlorite and singlet oxygen, and thereby provide a shield against several elements of phagocyte ROS killing [68]. GBS transcriptional regulators CovR [20] and CiaR [101] have also been linked to survive inside phagocytic cells, likely acting to coordinate expression of acid and stress survival genes.

### Bacterial translocation into the CNS

**Fig. 3:**
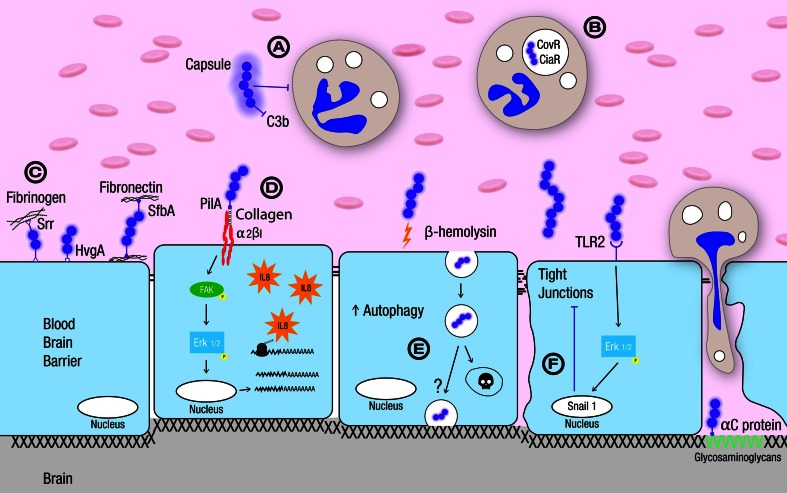
Group B Streptococcus interaction with the blood–brain barrier. **a** The GBS capsule promotes blood stream survival by preventing deposition of complement and ultimately phagocytosis. **b** GBS response regulators, CovR and CiaR, have been shown to further promote survival within phagocytic cells which will aid in GBS bloodstream survival. **c** GBS adhesins Srr, HvgA and SfbA promote GBS interaction with brain microvascular endothelial cells some by associating with extracellular matrix (ECM) components. **d** Another key GBS adhesin, the pilus tip protein PilA, binds collagen to bridge an interaction with α2β1 integrins on the endothelial cell surface. This initiates bacterial uptake and immune activation. **e** The GBS β-hemolysin activates brain microvascular endothelial cells including autophagy that may contribute to clearance of GBS by shuttling intracellular bacteria to the lysosome, although the exact mechanism of GBS transcytosis is unknown. **f** The host transcription factor, Snail1, which is a repressor of tight junctional components, is induced during GBS infection and results in the loss of tight junctions. This contributes to GBS penetration and BBB permeability during disease progression

Following bloodstream survival, GBS interacts directly with BBB endothelium, which can result in bacterial invasion of the BBB with subsequent infection of the CNS. This process can result from increased permeability of the BBB and/or the direct invasion of BMEC by the pathogen (Fig. 3). With the availability of in vitro tissue culture models of human BMEC (HBMEC) and animal models of GBS infection, significant progress has been made identifying and characterizing the molecular determinants that promote GBS–BBB interaction. GBS enter or “invade” brain endothelium apically and exit the cell on the basolateral side, thereby crossing the BBB transcellularly [90]. Electron microscopic (EM) studies have demonstrated the presence of the meningeal pathogen in membrane-bound vacuoles within HBMEC [23, 90], suggesting the involvement of endocytic pathways as well as avoidance of lysosomal fusion for BBB traversal. This process may be accomplished, at least in part, by tyrosine phosphorylation of focal adhesion kinase (FAK), which occurs upon GBS infection. Phosphorylation of FAK induces its association with PI3K and paxillin, an actin filament adaptor protein, and is required for efficient GBS HBMEC invasion.

To elucidate the GBS determinants involved in the pathogenesis of meningitis, many groups have focused on the characterization of serotype III, ST-17 GBS isolates responsible for CNS disease. Screening of a GBS ST-17 mutant library revealed a unique requirement for the novel “invasion associated gene”, *iagA*, in BBB penetration by GBS [29]. Decreased invasion of HBMEC by the GBS ∆*iagA* mutant in vitro was correlated with a reduced risk for development of meningitis and markedly diminished lethality in vivo. The *iagA* gene encodes an enzyme for biosynthesis of diglucosyldiacylglycerol, a membrane glycolipid that functions as an anchor for lipoteichoic acid (LTA), indicating that proper LTA anchoring is important to facilitate GBS BBB penetration [29]. Interestingly, clinical GBS isolates from infants with EoD or LoD possess higher quantities of cell-associated LTA than strains isolated from mucosal surfaces of asymptomatically colonized infants [89]. The availability of GBS genome sequences has enabled the identification of genes restricted to the ST-17 lineage. One gene, now called hypervirulent GBS adhesion (HvgA), was shown to be required for GBS hypervirulence [136]. GBS strains that express HvgA are more efficient in crossing the intestinal and blood–brain barriers in neonates, including choroid plexus epithelial cells and brain microvascular endothelium [136].

Proteins targeted for cell surface expression in GBS are predicted to share a C-terminal sequence (L/IPXTG) for sortase recognition and anchoring to the Gram-positive cell wall. In a paradigm-shifting study, it was discovered that GBS express cell wall-anchored pili [65]. Among the sequenced GBS genomes, two genetic loci encoding pili have been identified, Pilus Island (PI)-1 and PI-2, the second existing in one of two variants (PI-2a and PI-2b), and not all genomes contain both loci [73]. GBS PI-2a includes the genes encoding PilB, an LP(x)TG-motif-containing protein that polymerizes to form a pilus backbone, and accessory pilus proteins PilA and PilC that are incorporated in the pilus [73]. Both PilA and PilB promote adherence to and invasion of brain endothelium, respectively [74]. It has been demonstrated that PilA binds the extracellular matrix (ECM) component, collagen, and that collagen binding enhanced GBS attachment as well as uptake into HBMEC in a dose-dependent manner [4]. Further, the PilA–collagen complex engages α2-β1 integrins on brain endothelium to promote bacterial attachment and pro-inflammatory chemokine release [4]. As a result, increased neutrophil infiltration was correlated with increased BBB permeability and higher levels of bacterial CNS penetration in vivo [4].

In addition to PilA, other GBS factors interact with various ECM proteins and constituents to promote bacteria–BBB interactions. The GBS surface anchored alpha C protein (APC) was shown to interact directly with glucosaminoglycans (GAGs) on brain endothelium, and promote the establishment of GBS meningitis [15]. More recently, a GBS fibronectin-binding protein, Streptococcal fibronectin-binding factor A (SfbA), was shown to contribute to GBS invasion of HBMEC in vitro and to the development of meningitis in vivo [84]. Interestingly, studies have suggested that adherence to fibrinogen may be a general property of GBS to promote bloodstream survival and host cell interactions [120]. An important determinant recently implicated in fibrinogen binding and BBB interaction is the GBS serine-rich repeat (Srr) glycoprotein [120]. GBS strains carry 1 of 2 *srr* gene alleles, designated *srr1* and *srr2*, which are similar in architecture but show only limited homology (<20 % identity). Expression of the Srr-2 protein seems to be restricted to serotype III and ST-17 strains. Recent structural studies demonstrated that both Srr1 and Srr2 interact with tandem repeats of the fibrinogen Aα chain via a “dock, lock, and latch” mechanism [119]. Moreover, increased affinity between Srr2 and fibrinogen was observed, suggesting that a greater affinity for fibrinogen may contribute to the increased virulence associated with Srr2-expressing strains [119].

### Immune activation and inflammatory response in the brain

The host inflammatory response to GBS contributes significantly to the pathogenesis of meningitis and CNS injury. The first comprehensive microarray analysis of the BBB endothelium transcriptional response to a bacterial pathogen was examined during GBS infection [30]. Highly induced genes were those involved in the inflammatory response, including Interleukin (IL)-8, CXCL1, and CXCL2, ICAM-1, and GM-CSF, which function to orchestrate neutrophil recruitment, activation and enhanced survival [30]. Several studies have shown an association between leukocyte trafficking and BBB permeability and increased GBS penetration of the CNS, suggesting that PMN-mediated damage of the BBB has a significant role in the pathogenesis of GBS meningitis [4, 30]. It is clear that the GBS β-haemolysin/cytolysin (β-h/c) toxin contributes to immune activation and much of the observed disease pathology. Hemolysin expression has been shown to directly damage brain cells including brain endothelial cells [90], leptomeninges (meningioma cells) and astrocytes [2], and primary neurons [106]. Further, toxin expression was identified as a principal provocative factor for BBB activation, contributing to the development of meningitis [30]. Recently GBS β-h/c was also shown to activate autophagy in BBB endothelium [23]. Although results demonstrated that antibacterial autophagy provided a BBB cellular defense against invading and toxin producing bacteria, GBS was not completely eliminated, suggesting that GBS may actively thwart the autophagic pathway [23].

Microarray analysis of brain endothelium has also indicated that HBMEC respond to GBS infection by upregulating Snail1, a global transcriptional repressor of tight junction proteins [52]. Recent studies have demonstrated that during GBS infection transcript and protein levels of tight junction components ZO-1, Claudin-5 and Occludin were decreased in vitro in HBMEC and in vivo using murine and zebrafish models of GBS infection [52]. This was dependent on Snail1 induction, which was sufficient to facilitate tight junction disruption, promoting bacterial passage and disruption of the BBB [52]. Interestingly host integrins, ECM components and glycosaminoglycans involved in GBS–BBB interactions all preferentially localize to the basolateral surface of polarized endothelium. The subsequent loss of tight junctions may represent the critical first step to disrupting cell polarity that enables bacterial pathogens like GBS to engage basolaterally expressed host receptors and promote BBB permeability and progression to meningitis.

## *Streptococcus suis* meningitis

**Fig. 4:**
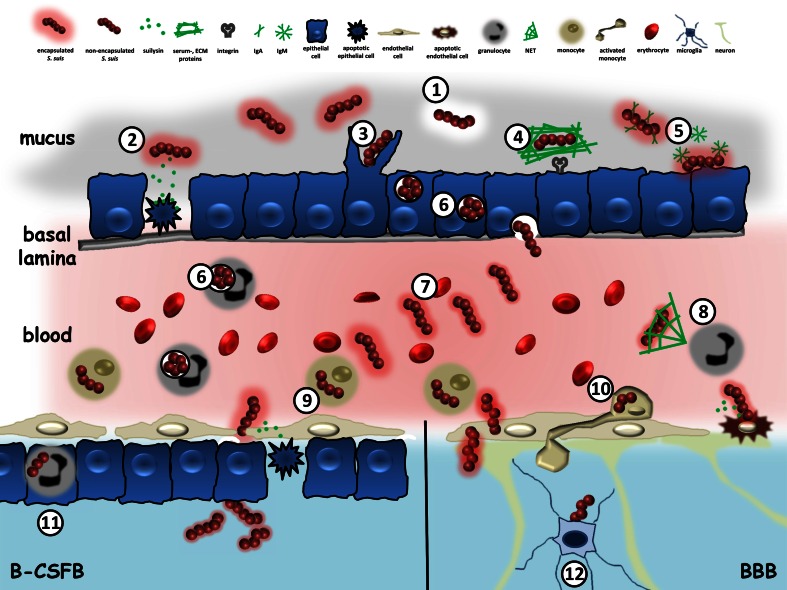
Pathogenesis of *Streptococcus suis* meningitis. *1* ApuA degrades glycogen and mediates adhesion to mucus. *2* *S. suis* harbors the cholesterol-dependent cytolysin SLY, which induces pore-formation in eukaryotic cells. *3* For a more effective adhesion and invasion, *S. suis* actively downregulates its polysaccharide capsule (CPS). *4* *S. suis* co-opts host proteins, such as serum and/or extracellular matrix (ECM) proteins and specifically interacts with epithelial cells by molecular bridges (e.g., with integrins). *5* *S. suis* evolves the proteases IGA1 and IdeSuis, which inactivate IgA and IgM, respectively, and thus prevents opsonization. *6* The Arginine Deiminase System (ADS) facilitates bacterial survival under acidic (intra-phagolysosomal) conditions in myeloid and non-myeloid cells. *7* CPS expression depends on nutrient availability and is high in blood but low in CSF. *8* Neutrophil Extracellular Trap (NET) formation is an ancient mechanism to combat bacterial infection. *S. suis* harbors to DNAses to circumvent NETosis. *9* *S. suis* uses monocytes to for dissemination. *10* *S. suis*-activated monocytes upregulate cellular adhesion molecules to interact with BMECs. *11* During infection, granulocytes overcome the B-CSFB by transmigration, thus serving as a vehicle for *S. suis* to disseminate into the CSF. *12* Upon *S. suis* infection, microglia upregulate innate immune pattern recognition receptors, such as TLR2, TLR3, CD14 and NOD2

*Streptococcus suis* is one of the most important porcine bacterial pathogens responsible for high economic losses in the swine industry worldwide. It causes a wide variety of diseases, including meningitis, septicaemia and endocarditis. Among the 33 serotypes originally described based on CPS antigens, serotype 2 is not only prevalent in swine diseases but is also considered to be an emerging zoonotic agent causing meningitis and streptococcal toxic shock-like syndrome in humans [42]. *S. suis* gained more attention since recent recognition of its high prevalence in human meningitis cases in South East and East Asia, and reports of outbreaks which resulted in high mortality rates [151]. Patients suffering from *S. suis* meningitis have cerebrospinal fluid with high numbers of neutrophils. One of the most striking sequel of *S. suis* meningitis is the establishment of deafness and/or vestibular dysfunction. In fact, the incidence of deafness following infection caused by this pathogen is consistently higher than that reported for other meningitis-causing bacteria, such as *S. pneumoniae*, *Neisseria meningitidis* and *Haemophilus influenza*. Following, host–pathogen interactions in the establishment of *S. suis* meningitis are summarized (depicted as a model in Fig. 4).

### Bacterial invasion and dissemination

As an opportunistic pathogen, *S. suis* colonizes the mucosal surfaces of the oropharyngeal and gastrointestinal tract of swine without inducing any clinical symptoms. However, since the mucosa constitutes a physical and immunological barrier to protect the host from invading pathogens, homeostasis between bacterium and host is a prerequisite for stable colonization. Additionally, inter- and intrabacterial competition for nutrients might also determine the success of an opportunist to permanently populate its preferred host. On the other hand, breakage of the epithelial barrier is often required for bacterial dissemination into deeper tissue sites. How *S. suis* interferes with the immune system of the mucosa and facilitates epithelial transmigration is only poorly characterized. Ferrando et al. [35] identified ApuA, an amylopullulanase with α(1,4)- and α(1,6)-glycolytic activity that allows *S. suis* to degrade glycogen and food-derived starch under in vivo conditions. Furthermore, ApuA mediates adhesion to mucus and, thus, displays an initial step in bacterial colonization. Immunoglobulins (Igs), such as IgA and IgM, are constituents of mucosal surfaces. By specifically coating the bacteria, Igs shape the microbiome and are involved in maintaining the bacteria–host homeostasis. *S. suis* has evolved two enzymes which specifically interact with mucosa-associated Igs. The IgA1 protease IGA is expressed in vivo and specifically cleaves IgA. Furthermore, its presence is strongly correlated with an invasive phenotype of *S. suis* suggesting an important role in pathogenesis [158]. Recently, the surface-associated IgM protease IdeSsuis was identified in a highly pathogenic serotype 2 strain. IdeSsuis specifically cleaves porcine IgM in vivo and, thereby, evades opsonization and complement-mediated killing when reaching the blood stream (see below) [115].

To reach systemic sites, *S. suis* has to breach the epithelial barrier. This may occur by different processes depending on expression of the CPS. Adhesion and invasion are significantly enhanced in unencapsulated isolates which is probably the result of a better accessibility of bacterial adhesins and invasins [8]. A variety of different bacterial cell-interacting proteins have been described (for review see [6]). Interestingly, in a recent study, Meng et al. [77] showed that capsule-dependent adhesion seems to be abrogated in co-infections of *S. suis* with highly pathogenic swine influenza virus. The underlying mechanisms, though not known in detail, might be based on different cellular receptor expression in complex primary multi-cellular precision-cut lung slices as compared to immortalized epithelial cell lines. In addition, interaction between the bacterium and the epithelial cell could also be of indirect nature. By co-opting host proteins of the extracellular matrix or serum proteins, *S. suis* is able to use them as a molecular bridge for adherence and invasion to/in host cells by receptor-mediated mechanisms (reviewed in [37]).

Epithelial transmigration might also be facilitated by cellular damage. *S. suis* possesses a thiol-activated cytolysin, suilysin (SLY), which can induce pore-formation in cholesterol-containing eukaryotic membranes. However, since bacterial mutants defective in SLY are still able to disseminate in the host [70], SLY activity seems to be important but not essential for systemic *S. suis* infections. Recently we discovered that SLY can promote adherence and host cell invasion of *S. suis* and that these effects also occurred at sublytic toxin concentrations [116]. However, the underlying mechanisms of SLY-mediated effects in adherence and invasion are yet unknown.

In the subepithelial environment, *S. suis* faces changing nutritional and immunological conditions. For example, whereas the capsule hinders bacterial adhesion (and invasion) to epithelial cells, it is essential for survival in blood due to its strong anti-phagocytic properties. Moreover, CPS mediates *S. suis* evasion of opsonization by immunoglobulins and activities of the complement system. Finally, a lower phagocytosis inevitably leads to reduced pro-inflammatory response and, thus, to a diminished tissue destruction and recruitment of immune cells. The fact that the capsule hinders transepithelial migration but enhances bacterial survival in the blood strongly indicates a tight regulation of CPS expression during pathogenesis. Indeed, Wu et al. [157] reported an increase in CPS expression when bacteria were grown in blood. In contrast, CPS gene transcription was low when *S. suis* was cultured in CSF, a compartment which is poor in nutrients. Accordingly, genes involved in carbohydrate and amino acid transport and metabolism were highly transcribed under such circumstances. Willenborg et al. [154] described a direct link between carbohydrate metabolism and CPS expression. A lack in the Carbon Control Protein A, the central regulator of Carbon Catabolite Repression in Gram-positive bacteria, led to a low capsule expression and attenuated survival in the presence of primary phagocytes [154]. Accordingly, other studies also revealed a link between nutrient starvation and enhanced virulence properties of *S. suis*. Thus, further work on metabolic adaptation will surely contribute to a better understanding of the pathogenesis of *S. suis* infections.

Similar to GBS, highly virulent and zoonotic serotype 2 *S. suis* strains possess neuraminidase activity to terminally link the CPS chains with sialic acid. However, in contrast to GBS, the sialic acid of *S. suis* is not α(2,3)-, but α(2,6)-linked to galactose moieties [145]. Whether this different sialyation pattern has an impact on immune recognition has to be proven in further studies. In addition to CPS expression and modification, other factors might be involved in survival in blood and bacterial dissemination. For example, modification of the bacterial cell wall by N-deacetylation of the peptidoglycan or D-alanylation of the lipoteichoic acid (LTA) leads to resistance against neutrophil-derived lysozyme and antimicrobial peptides [38, 39]. The generation of Neutrophil Extracellular Traps (NETs), an “ancient” antimicrobial mechanism of eukaryotic cells, is combated by *S. suis* with the expression of at least two different DNA-degrading enzymes. Consequently, inactivation of the extracellular *S. suis*-secreted nuclease A (SsnA) and the endonuclease A (EndAsuis) led to a reduced bacterial survival after co-cultivation with porcine granulocytes [25, 26]. Nevertheless, despite these anti-phagocytic factors, *S. suis* cannot prevent uptake by neutrophils. Eventually, some bacteria will be phagocytosed and inactivated in acidified phagolysosomes. However, *S. suis* also evolved strategies to overcome such inhospitable conditions. The pathogen possesses an Arginine Deiminase System (ADS), which increases intracellular survival of *S. suis* by neutralizing the intraphagolysomal pH [40]. The ADS is characterized as a metabolic enzymatic system, which catalyzes the degradation from arginine to ornithine and thereby producing ATP, citrulline, CO_2_ and NH_4_^+^. Thus, the ADS represents a multifunctional system important for bacterial metabolism and biological fitness in the host.

### Bacterial translocation into the CNS

*S. suis* bacteremia might result in the establishment of meningo-encephalitis in men and swine. However, to finally reach the cerebrospinal space or the brain parenchyma, respectively, *S. suis* is faced with two different cellular barriers, the BBB and the B-CSFB. The BBB is composed of a non-fenestrated monolayer of BMEC, which separates the brain from the intravascular space. BMECs are highly polarized with an apical and basolateral site expressing different surface proteins. This might be the reason why concordant in vitro studies revealed an effective adhesion but only a very low invasion of *S. suis* in porcine and human BMEC [7, 16]. The different kind of host cell interaction is further underlined by the fact that, in contrast to epithelial cells, the CPS seems to play only a minor role in the primary adhesion process [16]. Thus, alternative bacterial and/or cellular factors might be necessary to overcome the BBB. Nevertheless, similar to the interaction with epithelial cells, LPXTG-anchored surface proteins, lipoproteins as well as “moonlighting” proteins seem to be involved in binding and invasion of *S. suis* to BMEC to a certain extent (reviewed in [37]). BMEC respond to a *S. suis* infection by an upregulation of a variety of different cytokines and chemokines, such as IL-1, IL-6, IL-8, and TNFα [144]. Furthermore, Al-Numani et al. [1] showed an upregulation of the cellular adhesion molecules ICAM-1, CD11a/CD18 and CD11c/CD18 on human THP-1 monocytes upon *S. suis* infection. These stimulated monocytes exhibit a significantly increased adherence to endothelial cells, thus supporting the (modified) “Trojan horse” theory as a mechanism to overcome the BBB. However, although binding and invasion of *S. suis* to porcine monocytes was shown in vitro, in vivo evidence is still lacking.

In contrast to the BBB, the B-CSFB is a two-layer barrier made up of a fenestrated endothelium followed by the choroid plexus epithelial cells (CPEC). Significant work was done on the interaction of *S. suis* with human and porcine CPEC. Though it turned out that bacterial adhesion and invasion is similar to epithelial cells from other tissues, unique differences were observed in the preferred route of bacterial transmigration. *S. suis* adheres and invades CPEC significantly better when applied from the basolateral site than from the apical site [138]. This is most likely due to subcellular-specific receptor expression. Nevertheless, this in vitro phenotype reflects the in vivo situation where *S. suis* enters the cerebrospinal fluid from the blood via the plexus choroideus. The interaction of *S. suis* with CPEC goes along with distinct cellular and immunological responses. For example, infections with *S. suis* lead to rearrangements of tight junction proteins and induction of stress fiber formation, thus leading to a loss of barrier integrity and release of pro-inflammatory cytokines [137]. Expression of TNFα as well as cell adhesion molecules, such as VCAM-1 and ICAM-1, promotes adhesion and subsequent transmigration of PMNs through CPEC [152]. Interestingly, transmigration of PMNs occurs via the transcellular route. Since the authors also detected *S. suis* inside PMNs, the “Trojan horse” theory should be carefully revisited.

### Immune activation and inflammatory response in the brain

The pathogenesis of *S. suis* in the brain and its subsequent interactions with intracranial immune cells is only poorly understood. Dominguez-Punaro et al. [27] reported multifocal lesions from all areas of the brain as well as the meninges in mice upon *S. suis* infection. Lesions were accompanied by positive bacterial antigen reactions in immune-histochemical analysis and enhanced pro-inflammatory cytokine expression, which could later be reconstituted in vitro by infection of murine microglial cells with pathogenic serotype 2 *S. suis* [27, 28]. Interestingly, two independent studies reported an upregulation of innate immune pattern recognition receptors, such as TLR2, TLR3, CD14 and NOD2 in microglia upon *S. suis* infection [28, 160]. The mechanisms and functional relevance are unknown, but this may be a hint towards an intracellular fate of *S. suis*. It seems that *S. suis* does not actively invade astrocytes. However, a CPS- and SLY-dependent upregulation of pro-inflammatory cytokines in these cells was shown, a response that appears to be mainly TLR2 driven. Nevertheless, more detailed work is highly demanded to get better insights into the mechanism of *S. suis*–glial cell interactions.

## *Escherichia coli* K1-induced neonatal meningitis

*E. coli* K1 (*E. coli*) is the second leading cause of meningitis in neonates, but it is the leading pathogen in low-birth weight infants. Despite the drop in mortality rates from 50 % in 1970 to <20 % currently, the morbidity rates remain unchanged even with the use of effective antibiotics and supportive care [41]. The ever increasing numbers of antibiotic-resistant *E. coli* strains make the situation worrisome. An astounding 30–58 % of survivors suffer from serious neurological complications such as mental retardation, hearing loss and cortical blindness [41]. Although removal of bacteria from the circulation is the mainstay of antibiotic use, the release of large quantities of endotoxin from the dead bacteria triggers a massive inflammatory response resulting in septic shock. The use of corticosteroids to reduce this inflammatory response is ineffective in alleviating the neurological deficits associated with this disease. Therefore, a comprehensive understanding of the pathogenesis of *E. coli* meningitis is critical for the development of new therapeutic strategies.

Among *E. coli*, K1 CPS-decorated strains, a polymer of sialic acid residues, predominantly cause neonatal meningitis [54]. Besides K1 CPS, *E. coli* contains several surface structures such as pili, lipopolysaccharide, and outer membrane proteins that potentially interact with host tissues during the establishment of meningitis. Outer membrane protein A (OmpA) is the major protein of *E. coli,* and it is structurally conserved throughout the evolution [95]. However, recent studies have shown that pathogenic *E. coli* show minor differences in the extracellular loops of OmpA compared to non-pathogenic strains [127]. Several studies have demonstrated that OmpA plays a significant role in the pathogenesis of various diseases [62]. Other virulence factors of *E. coli* include IbeA, IbeB, yijiP, TraJ, aslA and cytotoxic necrotizing factor 1 (CNF-1) [53]. Here, we review the interactions of OmpA with various cells for binding to and invasion of *E. coli* and how they contribute to the pathogenesis of meningitis.

To gain insights into the pathophysiology of bacterial diseases, a careful selection and usage of animal models is clearly required. Newborn rat and mouse models have routinely used to study the pathogenesis of *E. coli*. These models mimic the human disease as they both depend on age for infection and cause the disease by hematogenous spread. The pathology of the brain in rats or mice is similar to infected humans showing edema, neutrophil infiltration, neuronal apoptosis and meningeal damage [80]. Therefore, the studies presented here are from in vitro experiments or very relevant newborn rat and mouse models.

### Bacterial invasion and dissemination

The colonization of mucosa by *E. coli* followed by invasion and crossing of the epithelial surfaces is critical for eventual spreading to intravascular space. Hek protein expressed by *E. coli* mediates adherence to and invasion of epithelial cells by binding to heparin sulfate glycosaminoglycans [34]. Succeeding invasion of mucosal surfaces allows *E. coli* to disseminate via hematogenous spread at which stage the bacterium must avoid initial serum bactericidal activity. Complement activation results in opsonization of bacteria for the formation of membrane attack complexes on the surface of pathogens, which mediates bacteriolysis. Opsonization with complement proteins also presents the bacteria to immune cells for phagocytosis. The K1 CPS of *E. coli* is shown to be necessary for the survival of the bacterium in the blood [54]. Subsequent studies using OmpA^−^*E. coli* additionally revealed that lack of OmpA renders the bacterium serum sensitive [97]. The bactericidal activity of serum against OmpA^−^*E. coli* appears to be mediated by classical complement pathway. Follow-up studies revealed that OmpA of *E. coli* binds to C4-binding protein (C4 bp), a classical complement pathway regulator to block the complement cascade reaction, and thereby avoids bacteriolysis and recognition by immune cells [97]. OmpA bound C4 bp acts as a co-factor for Factor I to cleave both C3b and C4b, which are important to present the bacterium to phagocytes [156].

The survival of *E. coli* in PMNs appears to be the first step in the pathogenic process as PMN depletion prevents the onset of meningitis in newborn mice [81]. The expression of OmpA is critical for survival inside PMNs after phagocytosis as OmpA^−^*E. coli* failed to survive. The phagocytosis of OmpA^−^*E. coli* by PMNs produces an enormous amount of reactive oxygen species (ROS) [122]. In contrast, OmpA^+^*E. coli* suppressed the release of ROS even in the presence of external stimuli such as LPS, indicating that *E. coli* overrides PMN machinery to prevent antimicrobial activity. Lack of other virulence factors such as S-fimbriae, IbeA, type-1 fimbriae and CNF-1 had no effect on the suppression of ROS production. Rac1, rac2 and gp91^Phox^, the components of NADPH oxidase, an enzyme complex required for the production of ROS, were suppressed by *E. coli* K1 at the transcriptional levels in PMNs [81].

Analysis of various surface receptors such as TLRs, Fc-gamma receptors and complement receptors on PMNs after infection with *E. coli* revealed that the bacterium increases the expression of gp96, an Hsp90 β-form but had no effect on other surface structures [81]. Again the OmpA of *E. coli* interacted with gp96 for entry and survival in PMNs, whereas, in the absence of gp96 expression, phagocytosed bacteria were killed efficiently. Moreover, entry of *E. coli* mediated by OmpA and gp96 interaction is required for reducing the levels of ROS. Substantiating the role of gp96 in *E. coli*-induced meningitis, suppression of gp96 using in vivo siRNA in three-day-old mice rendered them resistant to infection and prevented the brain damage. These gp96 knockdown mice could not develop bacteremia levels required to cross the BBB, suggesting that *E. coli* survival in PMNs is a critical step during the initial phases of infection.

**Fig. 5:**
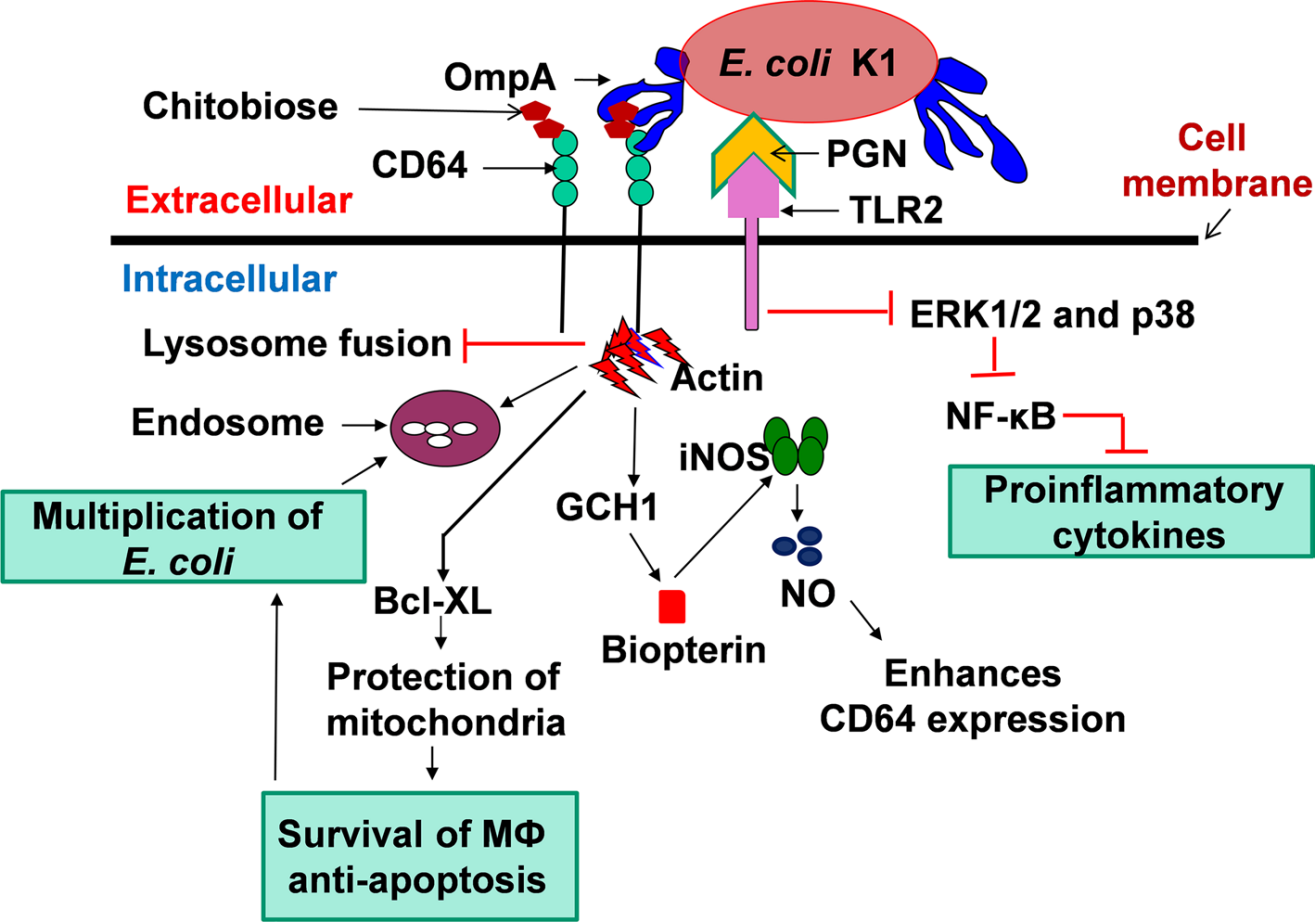
Mechanisms involved in *Escherichia coli* K1 manipulation of macrophages. The outer membrane protein A (OmpA) of *E. coli* K1 interacts with chitobiose moieties (GlcNAc1-4GlcNAc) in CD64 for inducing actin rearrangements to the sites of bacterial attachment for internalization of *E. coli*. During this process, the intracellular domain of CD64 triggers the upregulation of B cell lymphoma-extra large (Bcl-XL), an anti-apoptotic protein by an unknown mechanism to prevent apoptosis of the infected macrophages. In addition, toll-like receptor 2 (TLR2) ligands such as peptidoglycan (PGN) interaction with TLR2 also induces inducible nitric oxide (NO) production by activation of iNOS. Parallel to Bcl-XL upregulation, OmpA interaction with CD64 also enhances guanidine cyclohydrolase I (GCH1), which in turn produces biopterin. The biopterin subsequently acts as a co-factor for more inducible nitric oxide synthase (iNOS) activation and produce greater amounts of NO, which triggers the expression of CD64 to the cells surface. Thus, more *E. coli* bind to the receptor and enter the macrophages. The OmpA-CD64-mediated entry also avoids the fusion of lysosome with endosome, thereby finding a niche for survival and multiplication. To prevent the hostile conditions for bacterial survival, *E. coli* also suppresses mitogen-activated protein (MAP) kinases, extracellular signal-regulated kinases (ERK1/2), and p38, thereby the activity of nuclear factor-κB (NF-κB). This arm of signaling prevents the production of pro-inflammatory cytokines in macrophages. *Red lines* indicate inhibition of specific signaling pathway

Since PMNs are short-lived cells dying predominantly by apoptosis (Fig. 1b), *E. coli* must have alternative routes for survival and multiplication in neonates to reach high-grade bacteremia. Phagocytosis assays using RAW 264.7 and primary macrophages revealed that *E. coli* enters, survives and multiplies inside the cells, whereas OmpA^−^*E. coli* were killed by the cells immediately [134]. Of note, macrophage-depleted newborn mice became resistant to *E. coli* infection despite the presence of PMNs, suggesting that macrophages also provide a niche for bacterial multiplication. In macrophages, OmpA of *E. coli* binds to the alpha chain of Fc-gamma receptor I (CD64), the high-affinity IgG binding receptor via N-glycosylation sites [61]. Validating these studies, CD64^−/−^ newborn mice were resistant to *E. coli*-induced meningitis and adoptive transfer of wild-type macrophages into these mice sensitizes them to infection. Apoptosis of infected immune cells limits the dissemination of intracellular pathogens, thus preventing the spread of bacteria in the host. However, pathogens also developed strategies to manipulate the apoptotic mechanism in macrophages (Fig. 5). One such strategy *E. coli* utilized for an anti-apoptotic mechanism in macrophages was by increasing the expression of Bcl-XL, an anti-apoptotic protein [133]. OmpA^−^*E. coli,* on the other hand, enhanced the expression of Bax and Caspase 6 in infected macrophages, which eventually undergo apoptosis. *E. coli* infection of monocytes not only allows the bacteria to survive but also prevents the production of various cytokines and chemokines from the cells [118]. The blocking effect of pro-inflammatory cytokines by *E. coli* is due to the degradation of IκB followed by inhibition of NF-κB activity. Furthermore, *E. coli* controls ERK1/2 and p38 MAP kinases by modulating their phosphorylation status, and thus regulating IκB degradation. In that context, infection of three-day-old mice triggered the production of IL-10 at early stages of infection, indicating that suppression of pro-inflammatory response in replication stage is advantageous to *E. coli* for the establishment of meningitis [80]. Administration of a single dose of 5 µg of recombinant human IL-10 during bacteremia stages completely cleared the bacteria from the circulation and reversed sustained brain damage within four days post-infection.

### Bacterial translocation into the CNS

BMEC form the BBB that prevents the transport of harmful substances and pathogenic microorganisms from the blood to the brain. The development of high-grade bacteremia is a prerequisite for *E. coli* to interact with the BBB. All of the surface structures of *E. coli* K1 have potential to interact with BMEC for invasion and entry to the CNS. One of the surface appendages in *E. coli*, S-fimbriae (Sfa) that specifically interacts with NeuAcα2, 3Gal1, 3GlcNAc epitopes present on glycoproteins is shown to be responsible for binding to BMEC via SfaS adhesin present at the tip of Sfa [129]. However, Sfa plays no significant role in the invasion of HBMEC. Subsequent studies have demonstrated that type-1 fimbriae, which bind to mannose residues of glycoproteins, also contribute to the invasion of *E. coli* in HBMEC [139]. Nonetheless, when the type-1 fimbriae expression was similar to wild-type *E. coli* by keeping the *fimH* operon, which encodes the tip of type-1 fimbriae, in “ON” phase in an OmpA^−^*E. coli,* the bacterium could not invade. Furthermore, pretreatment of *E. coli* with α-methyl mannoside (an inhibitor of type-1 fimbriae) did not show any difference in the invasion, indicating that OmpA is the major determinant in *E. coli* invasion of HBMEC [63].

OmpA has been shown to bind to HBMEC for invasion via a lectin-like activity specific to GlcNAc1, 4GlcNAc (chitobiose) epitopes attached to asparagine-linked glycoproteins [99]. Corroborating the requirement of chitobiose moieties for the pathogenesis, treatment of *E. coli* with chitooligomers prior to infecting newborn rats prevented the occurrence of meningitis. Subsequent studies have identified a β-form of gp96, a heat-shock protein, present in HBMEC (designated as Ecgp96), which acts as a receptor for OmpA binding to and invasion of the cells. Ecgp96 is an 803 amino acid protein with a weak transmembrane domain [98]. The interaction of OmpA of *E. coli* with two N-glycosylation sites of Ecgp96 further enhances the expression of the receptor to which additional bacteria bind and invade HBMEC [63]. Additionally, the C-terminal domains of Ecgp96 are required for induction of signaling network to enter HBMEC [76]. *E. coli* interaction with HBMEC also triggers the expression of TLR2 at the surface, which forms a complex with Ecgp96 while OmpA^−^*E. coli* enhanced TLR4 expression and does not associate with the receptor [60]. Consistent with the requirement of TLR2 interaction with Ecgp96 TLR2^−/−^ newborn mice are resistant to infection while TLR4^−/−^ animals are very vulnerable to the development of meningitis.

For internalization, *E. coli* induces actin cytoskeletal rearrangements to trigger zipper-like mechanism in HBMEC, which engulfs the bacterium into the cell. Besides actin microfilaments, *E. coli* K1 also requires microtubules for invasion, which probably provides pulling force in HBMEC to internalize the bacteria. *E. coli* entry induces the phosphorylation of tyrosine residues of focal adhesion kinase (FAK), which is independent of Src kinase activity [105]. PI3-kinase activity is also critical for *E. coli* invasion of HBMEC, which subsequently activates PLCγ for the influx of extracellular calcium and mobilization of intracellular calcium [104, 130]. This calcium mobilization activates PKC-α, which interacts with caveolin-1, a 22 kDa protein present in caveolae of plasma membranes inducing the ingestion of *E. coli* by HBMEC [132]. Activated PKC-α associates with VE-cadherin, an adherens junction molecule, and releases β-catenin from the junctions, thereby increasing the permeability of HBMEC monolayers [131]. Pre-incubation of *E. coli* with anti-OmpA antibodies or HBMEC with anti-Ecgp96 antibodies decreased *E. coli*-induced permeability confirming that OmpA-Ecgp96 interaction is critical for tight junction disruption.

There is an ample evidence that nitric oxide (NO) acts as an antimicrobial molecule and a mediator of cerebral vascular permeability. *E. coli* upon invasion of HBMEC also produces higher amounts of NO by activating inducible nitric oxide synthase (iNOS) and generating cyclic GMP (cGMP), an important target downstream of NO [79]. Moreover, increased production of cGMP resulted in the activation of PKC-α, indicating that there might be two pools of PKC-α, one that is regulated by Ecgp96, and the second modulated by NO to enhance the permeability of HBMEC monolayers. Inhibition of iNOS by a specific inhibitor, aminoguanidine, prevented *E. coli* invasion by suppressing the expression of Ecgp96 [79]. Thus, inducible NO promotes *E. coli* invasion in HBMEC, unlike many other bacterial pathogens. Further studies have demonstrated that a rate limiting enzyme, GTP cyclohydrolase (GCH1), which produces a co-factor tetrahydrobiopterin required for iNOS activation, is also associated with intracellular Ecgp96 [121]. An inhibitor of GCH1, 2, 4-diamino hydroxyl pyrimidine (DAHP) pretreatment of HBMEC blocked the invasion in the cells. Both aminoguanidine and DAHP inhibited the onset of meningitis in 3-day-old mice by *E. coli*, highlighting the significance of NO production in the pathogenesis [79, 121]. In addition, screening of a small molecule library using HBMEC invasion assays recognized Telmisartan, an angiotensin II receptor 1 (AT1R) blocker as a potent inhibitor of invasion [64]. Follow-up experiments demonstrated that AT1R forms a complex with Ecgp96 during *E. coli* invasion of HBMEC. Newborn mice pretreated with TS are resistant to both the development of bacteremia and the entry of bacteria into the brain. These experiments clearly demonstrate that targeting Ecgp96 would be beneficial for averting *E. coli*-induced meningitis.

### Immune activation and inflammatory response in the brain

The survival and multiplication of *E. coli* in PMNs and macrophages result in the production of pro-inflammatory cytokines in the blood, which upregulates the expression of intracellular adhesion molecule 1 (ICAM-1) on the BBB. In addition, the interaction of OmpA of *E. coli* with Ecgp96 on HBMEC induces ICAM-1 expression, thereby enhancing the binding of THP-1 cells in culture [117]. This upregulation of ICAM-1 expression aids in the infiltration of PMNs during the onset of meningitis. Furthermore, gliosis and neuronal apoptosis in both cortex and hippocampus and the production of greater amounts of TNF-α and IL1β have been observed in the brains of newborn mice infected with *E. coli* [80]. Nonetheless, the interaction of *E. coli* with neuronal cells and glial cells is poorly studied. Further studies are clearly needed to gain a better understanding of whether the bacteria directly damages the brain or the damage is a causal effect of pro-inflammatory response.

## Conclusions and outlook

In summary, despite advances in antimicrobial therapy and vaccine development, bacterial meningitis represents a significant cause of morbidity and mortality, mainly in infants, children and in the elderly or immunocompromised patient. The emergence of antibiotic-resistant strains, e.g., *E. coli* and *S. pneumoniae*, phenotypic heterogeneity, e.g., meningococci, the lack of effective vaccines, e.g., GBS, or the occurrence of new emerging diseases as a results of zoonotic species jumps, e.g., *S. suis,* demands alternative strategies to prevent as many cases of bacterial meningitis and the associated neurological sequelae as possible. Significant progress has been made in identifying molecular mechanisms that contribute to host–pathogen interactions during the progression of CNS disease. Identification of common pathways employed by bacterial pathogens to breach mucosal barriers, survive in the blood stream and cross the BBB or B-CSF barrier will assist in the identification of important bacterial and host cell targets for the development of effective therapies. The identification of molecular patterns used by several bacterial species to cross the B-CSFB and BBB may lead to the systemic application of antibodies or antagonists blocking barrier epitopes involved in the attachment and transcytosis of bacteria. Vaccination against these bacterial patterns or prophylactic application of an antagonistic drug with low side effects can be an option, particularly in persons at a high risk of acquiring meningitis. Thus, targeting bacterial components and their associated signaling events should offer novel therapeutic strategies. A multi-disciplinary approach is necessary to incorporate all this knowledge into new testable hypotheses that will provide insight into the pathogenesis and pathophysiology of bacterial meningitis and the discovery of novel therapeutic and control strategies.

## References

[1] Al-Numani D, Segura M, Dore M, Gottschalk M (2003). Up-regulation of ICAM-1, CD11a/CD18 and CD11c/CD18 on human THP-1 monocytes stimulated by *Streptococcus suis* serotype 2. Clin Exp Immunol.

[2] Alkuwaity K, Taylor A, Heckels JE, Doran KS, Christodoulides M (2012). Group B Streptococcus interactions with human meningeal cells and astrocytes in vitro. PLoS One.

[3] Baker CJ, Edwards MS, Remington JS, Klein JO (2001). Group B streptococcal infections. Infectious diseases of the fetus and newborn infant.

[4] Banerjee A, Kim BJ, Carmona EM, Cutting AS, Gurney MA, Carlos C, Feuer R, Prasadarao NV, Doran KS (2011). Bacterial Pili exploit integrin machinery to promote immune activation and efficient blood-brain barrier penetration. Nat Commun.

[5] Barichello T, Collodel A, Generoso JS, Simoes LR, Moreira AP, Ceretta RA, Petronilho F, Quevedo J (2015). Targets for adjunctive therapy in pneumococcal meningitis. J Neuroimmunol.

[6] Baums CG, Valentin-Weigand P (2009). Surface-associated and secreted factors of *Streptococcus suis* in epidemiology, pathogenesis and vaccine development. Anim Health Res Rev/Conf Res Work Anim Dis.

[7] Benga L, Friedl P, Valentin-Weigand P (2005). Adherence of *Streptococcus suis* to porcine endothelial cells. J Vet Med B Infect Dis Vet Public Health.

[8] Benga L, Goethe R, Rohde M, Valentin-Weigand P (2004). Non-encapsulated strains reveal novel insights in invasion and survival of *Streptococcus suis* in epithelial cells. Cell Microbiol.

[9] Bergmann S, Lang A, Rohde M, Agarwal V, Rennemeier C, Grashoff C, Preissner KT, Hammerschmidt S (2009). Integrin-linked kinase is required for vitronectin-mediated internalization of *Streptococcus pneumoniae* by host cells. J Cell Sci.

[10] Berman PH, Banker BQ (1966). Neonatal meningitis—a clinical and pathological study of 29 cases. Pediatrics.

[11] Bernard SC, Simpson N, Join-Lambert O, Federici C, Laran-Chich MP, Maissa N, Bouzinba-Segard H, Morand PC, Chretien F, Taouji S, Chevet E, Janel S, Lafont F, Coureuil M, Segura A, Niedergang F, Marullo S, Couraud PO, Nassif X, Bourdoulous S (2014). Pathogenic *Neisseria meningitidis* utilizes CD147 for vascular colonization. Nat Med.

[12] Bradley CJ, Griffiths NJ, Rowe HA, Heyderman RS, Virji M (2005). Critical determinants of the interactions of capsule-expressing *Neisseria meningitidis* with host cells: the role of receptor density in increased cellular targeting via the outer membrane Opa proteins. Cell Microbiol.

[13] Brandt CT, Lundgren JD, Lund SP, Frimodt-Moller N, Christensen T, Benfield T, Espersen F, Hougaard DM, Ostergaard C (2004). Attenuation of the bacterial load in blood by pretreatment with granulocyte-colony-stimulating factor protects rats from fatal outcome and brain damage during *Streptococcus pneumoniae* meningitis. Infect Immun.

[14] Braun JS, Novak R, Herzog KH, Bodner SM, Cleveland JL, Tuomanen EI (1999). Neuroprotection by a caspase inhibitor in acute bacterial meningitis. Nat Med.

[15] Chang YC, Wang Z, Flax LA, Xu D, Esko JD, Nizet V, Baron MJ (2011). Glycosaminoglycan binding facilitates entry of a bacterial pathogen into central nervous systems. PLoS Pathog.

[16] Charland N, Nizet V, Rubens CE, Kim KS, Lacouture S, Gottschalk M (2000). *Streptococcus suis* serotype 2 interactions with human brain microvascular endothelial cells. Infect Immun.

[17] Coureuil M, Lecuyer H, Scott MG, Boularan C, Enslen H, Soyer M, Mikaty G, Bourdoulous S, Nassif X, Marullo S (2010). Meningococcus Hijacks a beta2-adrenoceptor/beta-Arrestin pathway to cross brain microvasculature endothelium. Cell.

[18] Coureuil M, Mikaty G, Miller F, Lecuyer H, Bernard C, Bourdoulous S, Dumenil G, Mege RM, Weksler BB, Romero IA, Couraud PO, Nassif X (2009). Meningococcal type IV pili recruit the polarity complex to cross the brain endothelium. Science.

[19] Coutinho LG, Grandgirard D, Leib SL, Agnez-Lima LF (2013). Cerebrospinal-fluid cytokine and chemokine profile in patients with pneumococcal and meningococcal meningitis. BMC Infect Dis.

[20] Cumley NJ, Smith LM, Anthony M, May RC (2012). The CovS/CovR acid response regulator is required for intracellular survival of group B Streptococcus in macrophages. Infect Immun.

[21] Cundell DR, Gerard NP, Gerard C, Idanpaan-Heikkila I, Tuomanen EI (1995). Streptococcus pneumoniae anchor to activated human cells by the receptor for platelet-activating factor. Nature.

[22] Cundell DR, Weiser JN, Shen J, Young A, Tuomanen EI (1995). Relationship between colonial morphology and adherence of *Streptococcus pneumoniae*. Infect Immun.

[23] Cutting AS, Del Rosario Y, Mu R, Rodriguez A, Till A, Subramani S, Gottlieb RA, Doran KS (2014). The role of autophagy during group B Streptococcus infection of blood-brain barrier endothelium. J Biol Chem.

[24] Dacey RG, Sande MA (1974). Effect of probenecid on cerebrospinal fluid concentrations of penicillin and cephalosporin derivatives. Antimicrob Agents Chemother.

[25] de Buhr N, Neumann A, Jerjomiceva N, von Kockritz-Blickwede M, Baums CG (2014). *Streptococcus suis* DNase SsnA contributes to degradation of neutrophil extracellular traps (NETs) and evasion of NET-mediated antimicrobial activity. Microbiology.

[26] de Buhr N, Stehr M, Neumann A, Naim HY, Valentin-Weigand P, von Kockritz-Blickwede M, Baums CG (2015). Identification of a novel DNase of *Streptococcus suis* (EndAsuis) important for neutrophil extracellular trap degradation during exponential growth. Microbiology.

[27] Dominguez-Punaro MC, Segura M, Plante MM, Lacouture S, Rivest S, Gottschalk M (2007). *Streptococcus suis* serotype 2, an important swine and human pathogen, induces strong systemic and cerebral inflammatory responses in a mouse model of infection. J Immunol.

[28] Dominguez-Punaro Mde L, Segura M, Contreras I, Lachance C, Houde M, Lecours MP, Olivier M, Gottschalk M (2010). In vitro characterization of the microglial inflammatory response to *Streptococcus suis*, an important emerging zoonotic agent of meningitis. Infect Immun.

[29] Doran KS, Engelson EJ, Khosravi A, Maisey HC, Fedtke I, Equils O, Michelsen KS, Arditi M, Peschel A, Nizet V (2005). Blood-brain barrier invasion by group B Streptococcus depends upon proper cell-surface anchoring of lipoteichoic acid. J Clin Investig.

[30] Doran KS, Liu GY, Nizet V (2003). Group B streptococcal beta-hemolysin/cytolysin activates neutrophil signaling pathways in brain endothelium and contributes to development of meningitis. J Clin Investig.

[31] Edmond KM, Kortsalioudaki C, Scott S, Schrag SJ, Zaidi AK, Cousens S, Heath PT (2012). Group B streptococcal disease in infants aged younger than 3 months: systematic review and meta-analysis. Lancet.

[32] Edwards MS, Rench MA, Haffar AA, Murphy MA, Desmond MM, Baker CJ (1985). Long-term sequelae of group B streptococcal meningitis in infants. J Pediatr.

[33] Eugene E, Hoffmann I, Pujol C, Couraud PO, Bourdoulous S, Nassif X (2002). Microvilli-like structures are associated with the internalization of virulent capsulated *Neisseria meningitidis* into vascular endothelial cells. J Cell Sci.

[34] Fagan RP, Lambert MA, Smith SG (2008). The hek outer membrane protein of *Escherichia coli* strain RS218 binds to proteoglycan and utilizes a single extracellular loop for adherence, invasion, and autoaggregation. Infect Immun.

[35] Ferrando ML, Fuentes S, de Greeff A, Smith H, Wells JM (2010). ApuA, a multifunctional alpha-glucan-degrading enzyme of *Streptococcus suis*, mediates adhesion to porcine epithelium and mucus. Microbiology.

[36] Fillon S, Soulis K, Rajasekaran S, Benedict-Hamilton H, Radin JN, Orihuela CJ, El Kasmi KC, Murti G, Kaushal D, Gaber MW, Weber JR, Murray PJ, Tuomanen EI (2006). Platelet-activating factor receptor and innate immunity: uptake of gram-positive bacterial cell wall into host cells and cell-specific pathophysiology. J Immunol.

[37] Fittipaldi N, Segura M, Grenier D, Gottschalk M (2012). Virulence factors involved in the pathogenesis of the infection caused by the swine pathogen and zoonotic agent *Streptococcus suis*. Future Microbiol.

[38] Fittipaldi N, Sekizaki T, Takamatsu D, de la Cruz Dominguez-Punaro M, Harel J, Bui NK, Vollmer W, Gottschalk M (2008). Significant contribution of the pgdA gene to the virulence of *Streptococcus suis*. Mol Microbiol.

[39] Fittipaldi N, Sekizaki T, Takamatsu D, Harel J, Dominguez-Punaro Mde L, Von Aulock S, Draing C, Marois C, Kobisch M, Gottschalk M (2008). D-Alanylation of lipoteichoic acid contributes to the virulence of *Streptococcus suis*. Infect Immun.

[40] Fulde M, Willenborg J, de Greeff A, Benga L, Smith HE, Valentin-Weigand P, Goethe R (2011). ArgR is an essential local transcriptional regulator of the arcABC operon in *Streptococcus suis* and is crucial for biological fitness in an acidic environment. Microbiology.

[41] Furyk JS, Swann O, Molyneux E (2011). Systematic review: neonatal meningitis in the developing world. Trop Med Int Health TM IH.

[42] Gottschalk M, Xu J, Calzas C, Segura M (2010). *Streptococcus suis*: a new emerging or an old neglected zoonotic pathogen?. Future Microbiol.

[43] Gould JM, Weiser JN (2002). The inhibitory effect of C-reactive protein on bacterial phosphorylcholine platelet-activating factor receptor-mediated adherence is blocked by surfactant. J Infect Dis.

[44] Grassme H, Becker KA (2013) Bacterial infections and ceramide. Handbook of experimental pharmacology. Springer-Verlag, Heidelberg, Germany, pp 305–320. doi:10.1007/978-3-7091-1511-4_15 10.1007/978-3-7091-1511-4_15 23563663

[45] Hoegen T, Tremel N, Klein M, Angele B, Wagner H, Kirschning C, Pfister HW, Fontana A, Hammerschmidt S, Koedel U (2011). The NLRP3 inflammasome contributes to brain injury in pneumococcal meningitis and is activated through ATP-dependent lysosomal cathepsin B release. J Immunol.

[46] Humphries HE, Triantafilou M, Makepeace BL, Heckels JE, Triantafilou K, Christodoulides M (2005). Activation of human meningeal cells is modulated by lipopolysaccharide (LPS) and non-LPS components of *Neisseria meningitidis* and is independent of Toll-like receptor (TLR)4 and TLR2 signalling. Cell Microbiol.

[47] Iovino F, Molema G, Bijlsma JJ (2014). Platelet endothelial cell adhesion molecule-1, a putative receptor for the adhesion of Streptococcus pneumoniae to the vascular endothelium of the blood-brain barrier. Infect Immun.

[48] Iovino F, Orihuela CJ, Moorlag HE, Molema G, Bijlsma JJE (2013). Interactions between blood-borne *Streptococcus pneumoniae* and the blood-brain barrier preceding meningitis. PloS One.

[49] Jen FE, Warren MJ, Schulz BL, Power PM, Swords WE, Weiser JN, Apicella MA, Edwards JL, Jennings MP (2013). Dual pili post-translational modifications synergize to mediate meningococcal adherence to platelet activating factor receptor on human airway cells. PLoS Pathog.

[50] Jones N, Bohnsack JF, Takahashi S, Oliver KA, Chan MS, Kunst F, Glaser P, Rusniok C, Crook DW, Harding RM, Bisharat N, Spratt BG (2003). Multilocus sequence typing system for group B streptococcus. J Clin Microbiol.

[51] Källström H, Liszewski MK, Atkinson JP, Jonsson AB (1997). Membrane cofactor protein (MCP or CD46) is a cellular pilus receptor for pathogenic Neisseria. Mol Microbiol.

[52] Kim BJ, Hancock BM, Bermudez A, Del Cid N, Reyes E, van Sorge NM, Lauth X, Smurthwaite CA, Hilton BJ, Stotland A, Banerjee A, Buchanan J, Wolkowicz R, Traver D, Doran KS (2015). Bacterial induction of Snail1 contributes to blood-brain barrier disruption. J Clin Investig.

[53] Kim KS (2003). Pathogenesis of bacterial meningitis: from bacteraemia to neuronal injury. Nat Rev Neurosci.

[54] Kim KS, Itabashi H, Gemski P, Sadoff J, Warren RL, Cross AS (1992). The K1-capsule is the critical determinant in the development of *Escherichia-coli* meningitis in the rat. J Clin Investig.

[55] Klein M, Paul R, Angele B, Popp B, Pfister HW, Koedel U (2006). Protein expression pattern in experimental pneumococcal meningitis. Microbes Infect/Inst Pasteur.

[56] Koedel U, Bernatowicz A, Paul R, Frei K, Fontana A, Pfister HW (1995). Experimental pneumococcal meningitis: cerebrovascular alterations, brain edema, and meningeal inflammation are linked to the production of nitric oxide. Ann Neurol.

[57] Koedel U, Frankenberg T, Kirschnek S, Obermaier B, Hacker H, Paul R, Hacker G (2009). Apoptosis is essential for neutrophil functional shutdown and determines tissue damage in experimental pneumococcal meningitis. PLoS Pathog.

[58] Koppe U, Suttorp N, Opitz B (2012). Recognition of *Streptococcus pneumoniae* by the innate immune system. Cell Microbiol.

[59] Kostyukova NN, Volkova MO, Ivanova VV, Kvetnaya AS (1995). A study of pathogenic factors of *Streptococcus pneumoniae* strains causing meningitis. FEMS Immunol Med Microbiol.

[60] Krishnan S, Chen S, Turcatel G, Arditi M, Prasadarao NV (2013). Regulation of Toll-like receptor 2 interaction with Ecgp96 controls *Escherichia coli* K1 invasion of brain endothelial cells. Cell Microbiol.

[61] Krishnan S, Liu F, Abrol R, Hodges J, Goddard WA, Prasadarao NV (2014). The interaction of N-glycans in Fc gamma Receptor I alpha-chain with *Escherichia coli* K1 outer membrane protein A for entry into macrophages experimental and computational analysis. J Biol Chem.

[62] Krishnan S, Prasadarao NV (2012). Outer membrane protein A and OprF: versatile roles in Gram-negative bacterial infections. FEBS J.

[63] Krishnan S, Prasadarao NV (2014). Identification of minimum carbohydrate moiety in N-glycosylation sites of brain endothelial cell glycoprotein 96 for interaction with *Escherichia coli* K1 outer membrane protein A. Microbes Infect/Inst Pasteur.

[64] Krishnan S, Shanmuganathan MV, Behenna D, Stoltz BM, Prasadarao NV (2014). Angiotensin II receptor type 1—a novel target for preventing neonatal meningitis in mice by *Escherichia coli* K1. J Infect Dis.

[65] Lauer P, Rinaudo CD, Soriani M, Margarit I, Maione D, Rosini R, Taddei AR, Mora M, Rappuoli R, Grandi G, Telford JL (2005). Genome analysis reveals pili in Group B Streptococcus. Science.

[66] Leib SL, Heimgartner C, Bifrare YD, Loeffler JM, Taauber MG (2003). Dexamethasone aggravates hippocampal apoptosis and learning deficiency in pneumococcal meningitis in infant rats. Pediatr Res.

[67] Leib SL, Leppert D, Clements J, Tauber MG (2000). Matrix metalloproteinases contribute to brain damage in experimental pneumococcal meningitis. Infect Immun.

[68] Liu GY, Doran KS, Lawrence T, Turkson N, Puliti M, Tissi L, Nizet V (2004). Sword and shield: linked group B streptococcal beta-hemolysin/cytolysin and carotenoid pigment function to subvert host phagocyte defense. Proc Natl Acad Sci USA.

[69] Liu X, Chauhan VS, Young AB, Marriott I (2010). NOD2 mediates inflammatory responses of primary murine glia to *Streptococcus pneumoniae*. Glia.

[70] Lun S, Perez-Casal J, Connor W, Willson PJ (2003). Role of suilysin in pathogenesis of *Streptococcus suis* capsular serotype 2. Microb Pathog.

[71] Mahdi LK, Wang H, Van der Hoek MB, Paton JC, Ogunniyi AD (2012). Identification of a novel pneumococcal vaccine antigen preferentially expressed during meningitis in mice. J Clin Investig.

[72] Mairey E, Genovesio A, Donnadieu E, Bernard C, Jaubert F, Pinard E, Seylaz J, Olivo-Marin JC, Nassif X, Dumenil G (2006). Cerebral microcirculation shear stress levels determine *Neisseria meningitidis* attachment sites along the blood-brain barrier. J Exp Med.

[73] Maisey HC, Doran KS, Nizet V (2008). Recent advances in understanding the molecular basis of group B Streptococcus virulence. Expert Rev Mol Med.

[74] Maisey HC, Hensler M, Nizet V, Doran KS (2007). Group B streptococcal pilus proteins contribute to adherence to and invasion of brain microvascular endothelial cells. J Bacteriol.

[75] Mann B, Thornton J, Heath R, Wade KR, Tweten RK, Gao G, El Kasmi K, Jordan JB, Mitrea DM, Kriwacki R, Maisonneuve J, Alderson M, Tuomanen EI (2014). Broadly protective protein-based pneumococcal vaccine composed of pneumolysin toxoid-CbpA peptide recombinant fusion protein. J Infect Dis.

[76] Maruvada R, Argon Y, Prasadarao NV (2008). *Escherichia coli* interaction with human brain microvascular endothelial cells induces signal transducer and activator of transcription 3 association with the C-terminal domain of Ec-gp96, the outer membrane protein A receptor for invasion. Cell Microbiol.

[77] Meng F, Wu NH, Nerlich A, Herrler G, Valentin-Weigand P, Seitz M (2015). Dynamic virus-bacterium interactions in a porcine precision-cut lung slice coinfection model: swine influenza virus paves the way for *streptococcus suis* infection in a two-step process. Infect Immun.

[78] Mitchell L, Smith SH, Braun JS, Herzog KH, Weber JR, Tuomanen EI (2004). Dual phases of apoptosis in pneumococcal meningitis. J Infect Dis.

[79] Mittal R, Gonzalez-Gomez I, Goth KA, Prasadarao NV (2010). Inhibition of inducible nitric oxide controls pathogen load and brain damage by enhancing phagocytosis of *Escherichia coli* K1 in neonatal meningitis. Am J Pathol.

[80] Mittal R, Gonzalez-Gomez I, Panigrahy A, Goth K, Bonnet R, Prasadarao NV (2010). IL-10 administration reduces PGE-2 levels and promotes CR3-mediated clearance of Escherichia coli K1 by phagocytes in meningitis. J Exp Med.

[81] Mittal R, Prasadarao NV (2011). gp96 expression in neutrophils is critical for the onset of *Escherichia coli* K1 (RS218) meningitis. Nature Commun.

[82] Mook-Kanamori BB, Geldhoff M, van der Poll T, van de Beek D (2011). Pathogenesis and pathophysiology of pneumococcal meningitis. Clin Microbiol Rev.

[83] Moxon ER, Ostrow PT (1977). *Haemophilus influenzae* meningitis in infant rats: role of bacteremia in pathogenesis of age-dependent inflammatory responses in cerebrospinal fluid. J Infect Dis.

[84] Mu R, Kim BJ, Paco C, Del Rosario Y, Courtney HS, Doran KS (2014). Identification of a group B streptococcal fibronectin binding protein, SfbA, that contributes to invasion of brain endothelium and development of meningitis. Infect Immun.

[85] Nassif X, Bourdoulous S, Eugene E, Couraud PO (2002). How do extracellular pathogens cross the blood-brain barrier?. Trends Microbiol.

[86] Nau R, Bruck W (2002). Neuronal injury in bacterial meningitis: mechanisms and implications for therapy. Trends Neurosci.

[87] Nau R, Gerber J, Bunkowski S, Bruck W (2004). Axonal injury, a neglected cause of CNS damage in bacterial meningitis. Neurology.

[88] Nau R, Soto A, Bruck W (1999). Apoptosis of neurons in the dentate gyrus in humans suffering from bacterial meningitis. J Neuropathol Exp Neurol.

[89] Nealon TJ, Mattingly SJ (1983). Association of elevated levels of cellular lipoteichoic acids of group B streptococci with human neonatal disease. Infect Immun.

[90] Nizet V, Kim KS, Stins M, Jonas M, Chi EY, Nguyen D, Rubens CE (1997). Invasion of brain microvascular endothelial cells by group B streptococci. Infect Immun.

[91] Orihuela CJ, Mahdavi J, Thornton J, Mann B, Wooldridge KG, Abouseada N, Oldfield NJ, Self T, Ala’Aldeen DA, Tuomanen EI (2009). Laminin receptor initiates bacterial contact with the blood brain barrier in experimental meningitis models. J Clin Investig.

[92] Ostergaard C, Benfield T, Gesser B, Kharazmi A, Frimodt-Moller N, Espersen F, Lundgren JD (1999). Pretreatment with granulocyte colony-stimulating factor attenuates the inflammatory response but not the bacterial load in cerebrospinal fluid during experimental pneumococcal meningitis in rabbits. Infect Immun.

[93] Patterson H, Saralahti A, Parikka M, Dramsi S, Trieu-Cuot P, Poyart C, Rounioja S, Ramet M (2012). Adult zebrafish model of bacterial meningitis in *Streptococcus agalactiae* infection. Dev Comp Immunol.

[94] Paul R, Koedel U, Pfister HW (2003). Using knockout mice to study experimental meningitis. Arch Immunol Ther Exp.

[95] Pautsch A, Schulz GE (2000). High-resolution structure of the OmpA membrane domain. J Mol Biol.

[96] Petersdorf RG, Swarner DR, Garcia M (1962). Studies on the pathogenesis of meningitis. II. Development of meningitis during pneumococcal bacteremia. J Clin Investig.

[97] Prasadarao NV, Blom AM, Villoutreix BO, Linsangan LC (2002). A novel interaction of outer membrane protein A with C4b binding protein mediates serum resistance of *Escherichia coli* K1. J Immunol.

[98] Prasadarao NV, Srivastava PK, Rudrabhatla RS, Kim KS, Huang SH, Sukumaran SK (2003). Cloning and expression of the *Escherichia coli* K1 outer membrane protein A receptor, a gp96 homologue. Infect Immun.

[99] Prasadarao NV, Wass CA, Kim KS (1996). Endothelial cell GlcNAc beta 1-4GlcNAc epitopes for outer membrane protein A enhance traversal of *Escherichia coli* across the blood-brain barrier. Infect Immun.

[100] Pron B, Taha MK, Rambaud C, Fournet JC, Pattey N, Monnet JP, Musilek M, Beretti JL, Nassif X (1997). Interaction of *Neisseria meningitidis* with the components of the blood-brain barrier correlates with an increased expression of PilC. J Infect Dis.

[101] Quach D, van Sorge NM, Kristian SA, Bryan JD, Shelver DW, Doran KS (2009). The CiaR response regulator in group B Streptococcus promotes intracellular survival and resistance to innate immune defenses. J Bacteriol.

[102] Quirante J, Ceballos R, Cassady G (1974). Group B beta-hemolytic streptococcal infection in the newborn. I. Early onset infection. Am J Dis Child.

[103] Radin JN, Orihuela CJ, Murti G, Guglielmo C, Murray PJ, Tuomanen EI (2005). Beta-arrestin 1 participates in platelet-activating factor receptor-mediated endocytosis of *Streptococcus pneumoniae*. Infect Immun.

[104] Reddy MA, Prasadarao NV, Wass CA, Kim KS (2000). Phosphatidylinositol 3-kinase activation and interaction with focal adhesion kinase in *Escherichia coli* K1 invasion of human brain microvascular endothelial cells. J Biol Chem.

[105] Reddy MA, Wass CA, Kim KS, Schlaepfer DD, Prasadarao NV (2000). Involvement of focal adhesion kinase in *Escherichia coli* invasion of human brain microvascular endothelial cells. Infect Immun.

[106] Reiss A, Braun JS, Jager K, Freyer D, Laube G, Buhrer C, Felderhoff-Muser U, Stadelmann C, Nizet V, Weber JR (2011). Bacterial pore-forming cytolysins induce neuronal damage in a rat model of neonatal meningitis. J Infect Dis.

[107] Ring A, Weiser JN, Tuomanen EI (1998). Pneumococcal trafficking across the blood-brain barrier. Molecular analysis of a novel bidirectional pathway. J Clin Investig.

[108] Rosenstein NE, Perkins BA, Stephens DS, Popovic T, Hughes JM (2001). Meningococcal disease. N Engl J Med.

[109] Rubens CE, Wessels MR, Heggen LM, Kasper DL (1987). Transposon mutagenesis of type III group B Streptococcus: correlation of capsule expression with virulence. Proc Natl Acad Sci USA.

[110] Sa ECC, Griffiths NJ, Virji M (2010). *Neisseria meningitidis* Opc invasin binds to the sulphated tyrosines of activated vitronectin to attach to and invade human brain endothelial cells. PLoS Pathog.

[111] Sadarangani M, Pollard AJ, Gray-Owen SD (2011). Opa proteins and CEACAMs: pathways of immune engagement for pathogenic Neisseria. FEMS Microbiol Rev.

[112] Sarkari J, Pandit N, Moxon ER, Achtman M (1994). Variable expression of the Opc outer membrane protein in *Neisseria meningitidis* is caused by size variation of a promoter containing poly-cytidine. Mol Microbiol.

[113] Scheld WM, Koedel U, Nathan B, Pfister HW (2002). Pathophysiology of bacterial meningitis: mechanism(s) of neuronal injury. J Infect Dis.

[114] Schubert-Unkmeir A, Konrad C, Slanina H, Czapek F, Hebling S, Frosch M (2010). *Neisseria meningitidis* induces brain microvascular endothelial cell detachment from the matrix and cleavage of occludin: a role for MMP-8. PLoS Pathog.

[115] Seele J, Beineke A, Hillermann LM, Jaschok-Kentner B, von Pawel-Rammingen U, Valentin-Weigand P, Baums CG (2015). The immunoglobulin M-degrading enzyme of *Streptococcus suis*, IdeSsuis, is involved in complement evasion. Vet Res.

[116] Seitz M, Baums CG, Neis C, Benga L, Fulde M, Rohde M, Goethe R, Valentin-Weigand P (2013). Subcytolytic effects of suilysin on interaction of *Streptococcus suis* with epithelial cells. Vet Microbiol.

[117] Selvaraj SK, Periandythevar P, Prasadarao NV (2007). Outer membrane protein A of *Escherichia coli* K1 selectively enhances the expression of intercellular adhesion molecule-1 in brain microvascular endothelial cells. Microbes Infect/Inst Pasteur.

[118] Selvaraj SK, Prasadarao NV (2005). *Escherichia coli* K1 inhibits proinflammatory cytokine induction in monocytes by preventing NF-kappaB activation. J Leukoc Biol.

[119] Seo HS, Minasov G, Seepersaud R, Doran KS, Dubrovska I, Shuvalova L, Anderson WF, Iverson TM, Sullam PM (2013). Characterization of fibrinogen binding by glycoproteins Srr1 and Srr2 of *Streptococcus agalactiae*. J Biol Chem.

[120] Seo HS, Mu R, Kim BJ, Doran KS, Sullam PM (2012). Binding of glycoprotein Srr1 of *Streptococcus agalactiae* to fibrinogen promotes attachment to brain endothelium and the development of meningitis. PLoS Pathog.

[121] Shanmuganathan MV, Krishnan S, Fu X, Prasadarao NV (2013). Attenuation of biopterin synthesis prevents *Escherichia coli* K1 invasion of brain endothelial cells and the development of meningitis in newborn mice. J Infect Dis.

[122] Shanmuganathan MV, Krishnan S, Fu X, Prasadarao NV (2014). *Escherichia coli* K1 induces pterin production for enhanced expression of Fcgamma receptor I to invade RAW 264.7 macrophages. Microbes Infect/Inst Pasteur.

[123] Simonis A, Hebling S, Gulbins E, Schneider-Schaulies S, Schubert-Unkmeir A (2014). Differential activation of acid sphingomyelinase and ceramide release determines invasiveness of *Neisseria meningitidis* into brain endothelial cells. PLoS Pathog.

[124] Slanina H, Hebling S, Hauck CR, Schubert-Unkmeir A (2012). Cell invasion by *Neisseria meningitidis* requires a functional interplay between the focal adhesion kinase, Src and cortactin. PloS One.

[125] Slanina H, Konig A, Hebling S, Hauck CR, Frosch M, Schubert-Unkmeir A (2010). Entry of *Neisseria meningitidis* into mammalian cells requires the Src family protein tyrosine kinases. Infect Immun.

[126] Slanina H, Mundlein S, Hebling S, Schubert-Unkmeir A (2014). Role of epidermal growth factor receptor signaling in the interaction of *Neisseria meningitidis* with endothelial cells. Infect Immun.

[127] Smith SG, Mahon V, Lambert MA, Fagan RP (2007). A molecular Swiss army knife: OmpA structure, function and expression. FEMS Microbiol Lett.

[128] Sokolova O, Heppel N, Jagerhuber R, Kim KS, Frosch M, Eigenthaler M, Schubert-Unkmeir A (2004). Interaction of *Neisseria meningitidis* with human brain microvascular endothelial cells: role of MAP- and tyrosine kinases in invasion and inflammatory cytokine release. Cell Microbiol.

[129] Stins MF, Prasadarao NV, Ibric L, Wass CA, Luckett P, Kim KS (1994). Binding characteristics of S fimbriated *Escherichia coli* to isolated brain microvascular endothelial cells. Am J Pathol.

[130] Sukumaran SK, McNamara G, Prasadarao NV (2003). *Escherichia coli* K-1 interaction with human brain micro-vascular endothelial cells triggers phospholipase C-gamma1 activation downstream of phosphatidylinositol 3-kinase. J Biol Chem.

[131] Sukumaran SK, Prasadarao NV (2003). *Escherichia coli* K1 invasion increases human brain microvascular endothelial cell monolayer permeability by disassembling vascular-endothelial cadherins at tight junctions. J Infect Dis.

[132] Sukumaran SK, Quon MJ, Prasadarao NV (2002). *Escherichia coli* K1 internalization via caveolae requires caveolin-1 and protein kinase Calpha interaction in human brain microvascular endothelial cells. J Biol Chem.

[133] Sukumaran SK, Selvaraj SK, Prasadarao NV (2004). Inhibition of apoptosis by *Escherichia coli* K1 is accompanied by increased expression of BClXL and blockade of mitochondrial cytochrome c release in macrophages. Infect Immun.

[134] Sukumaran SK, Shimada H, Prasadarao NV (2003). Entry and intracellular replication of *Escherichia coli* K1 in macrophages require expression of outer membrane protein A. Infect Immun.

[135] Tauber MG, Kennedy SL, Tureen JH, Lowenstein DH (1992). Experimental pneumococcal meningitis causes central nervous system pathology without inducing the 72-kd heat shock protein. Am J Pathol.

[136] Tazi A, Disson O, Bellais S, Bouaboud A, Dmytruk N, Dramsi S, Mistou MY, Khun H, Mechler C, Tardieux I, Trieu-Cuot P, Lecuit M, Poyart C (2010). The surface protein HvgA mediates group B streptococcus hypervirulence and meningeal tropism in neonates. J Exp Med.

[137] Tenenbaum T, Matalon D, Adam R, Seibt A, Wewer C, Schwerk C, Galla HJ, Schroten H (2008). Dexamethasone prevents alteration of tight junction-associated proteins and barrier function in porcine choroid plexus epithelial cells after infection with *Streptococcus suis* in vitro. Brain Res.

[138] Tenenbaum T, Papandreou T, Gellrich D, Friedrichs U, Seibt A, Adam R, Wewer C, Galla HJ, Schwerk C, Schroten H (2009). Polar bacterial invasion and translocation of *Streptococcus suis* across the blood-cerebrospinal fluid barrier in vitro. Cell Microbiol.

[139] Teng CH, Cai M, Shin S, Xie Y, Kim KJ, Khan NA, Di Cello F, Kim KS (2005). *Escherichia coli* K1 RS218 interacts with human brain microvascular endothelial cells via type 1 fimbria bacteria in the fimbriated state. Infect Immun.

[140] Tibussek D, Sinclair A, Yau I, Teatero S, Fittipaldi N, Richardson SE, Mayatepek E, Jahn P, Askalan R (2015). Late-onset group B streptococcal meningitis has cerebrovascular complications. J Pediatr.

[141] Tuomanen E, Liu H, Hengstler B, Zak O, Tomasz A (1985). The induction of meningeal inflammation by components of the pneumococcal cell wall. J Infect Dis.

[142] Uchiyama S, Carlin AF, Khosravi A, Weiman S, Banerjee A, Quach D, Hightower G, Mitchell TJ, Doran KS, Nizet V (2009). The surface-anchored NanA protein promotes pneumococcal brain endothelial cell invasion. J Exp Med.

[143] Unkmeir A, Latsch K, Dietrich G, Wintermeyer E, Schinke B, Schwender S, Kim KS, Eigenthaler M, Frosch M (2002). Fibronectin mediates Opc-dependent internalization of *Neisseria meningitidis* in human brain microvascular endothelial cells. Mol Microbiol.

[144] Vadeboncoeur N, Segura M, Al-Numani D, Vanier G, Gottschalk M (2003). Pro-inflammatory cytokine and chemokine release by human brain microvascular endothelial cells stimulated by *Streptococcus suis* serotype 2. FEMS Immunol Med Microbiol.

[145] Van Calsteren MR, Gagnon F, Lacouture S, Fittipaldi N, Gottschalk M (2010). Structure determination of *Streptococcus suis* serotype 2 capsular polysaccharide. Biochem cell Biol Biochim Biol Cellulaire.

[146] van Ginkel FW, McGhee JR, Watt JM, Campos-Torres A, Parish LA, Briles DE (2003). Pneumococcal carriage results in ganglioside-mediated olfactory tissue infection. Proc Natl Acad Sci USA.

[147] van Putten JP, Paul SM (1995). Binding of syndecan-like cell surface proteoglycan receptors is required for *Neisseria gonorrhoeae* entry into human mucosal cells. The EMBO J.

[148] Virji M (2009). Pathogenic neisseriae: surface modulation, pathogenesis and infection control. Nat Rev Microbiol.

[149] Waage A, Brandtzaeg P, Halstensen A, Kierulf P, Espevik T (1989). The complex pattern of cytokines in serum from patients with meningococcal septic shock. Association between interleukin 6, interleukin 1, and fatal outcome. J Exp Med.

[150] Watt JP, Wolfson LJ, O’Brien KL, Henkle E, Deloria-Knoll M, McCall N, Lee E, Levine OS, Hajjeh R, Mulholland K, Cherian T, Hib, Pneumococcal Global Burden of Disease Study T (2009) Burden of disease caused by *Haemophilus influenzae* type b in children younger than 5 years: global estimates. Lancet 374:903–911. doi:10.1016/S0140-6736(09)61203-4 10.1016/S0140-6736(09)61203-4 19748399

[151] Wertheim HF, Nghia HD, Taylor W, Schultsz C (2009). *Streptococcus suis*: an emerging human pathogen. Clin Infect Dis Off Publ Infect Dis Soc Am.

[152] Wewer C, Seibt A, Wolburg H, Greune L, Schmidt MA, Berger J, Galla HJ, Quitsch U, Schwerk C, Schroten H, Tenenbaum T (2011). Transcellular migration of neutrophil granulocytes through the blood-cerebrospinal fluid barrier after infection with *Streptococcus suis*. J Neuroinflamm.

[153] Whalen CM, Hockin JC, Ryan A, Ashton F (1995). The changing epidemiology of invasive meningococcal disease in Canada, 1985 through 1992. Emergence of a virulent clone of *Neisseria meningitidis*. JAMA.

[154] Willenborg J, Fulde M, de Greeff A, Rohde M, Smith HE, Valentin-Weigand P, Goethe R (2011). Role of glucose and CcpA in capsule expression and virulence of *Streptococcus suis*. Microbiology.

[155] Wippel C, Maurer J, Fortsch C, Hupp S, Bohl A, Ma J, Mitchell TJ, Bunkowski S, Bruck W, Nau R, Iliev AI (2013). Bacterial cytolysin during meningitis disrupts the regulation of glutamate in the brain, leading to synaptic damage. PLoS Pathog.

[156] Wooster DG, Maruvada R, Blom AM, Prasadarao NV (2006). Logarithmic phase *Escherichia coli* K1 efficiently avoids serum killing by promoting C4 bp-mediated C3b and C4b degradation. Immunology.

[157] Wu Z, Wu C, Shao J, Zhu Z, Wang W, Zhang W, Tang M, Pei N, Fan H, Li J, Yao H, Gu H, Xu X, Lu C (2014). The *Streptococcus suis* transcriptional landscape reveals adaptation mechanisms in pig blood and cerebrospinal fluid. RNA.

[158] Zhang A, Mu X, Chen B, Liu C, Han L, Chen H, Jin M (2010). Identification and characterization of IgA1 protease from *Streptococcus suis*. Vet Microbiol.

[159] Zhang JR, Mostov KE, Lamm ME, Nanno M, Shimida S, Ohwaki M, Tuomanen E (2000). The polymeric immunoglobulin receptor translocates pneumococci across human nasopharyngeal epithelial cells. Cell.

[160] Zheng H, Sun H, Dominguez-Punaro Mde L, Bai X, Ji S, Segura M, Xu J (2013). Evaluation of the pathogenesis of meningitis caused by *Streptococcus suis* sequence type 7 using the infection of BV2 microglial cells. J Med Microbiol.

[161] Zysk G, Bruck W, Gerber J, Bruck Y, Prange HW, Nau R (1996). Anti-inflammatory treatment influences neuronal apoptotic cell death in the dentate gyrus in experimental pneumococcal meningitis. J Neuropathol Exp Neurol.

[162] Zysk G, Schneider-Wald BK, Hwang JH, Bejo L, Kim KS, Mitchell TJ, Hakenbeck R, Heinz HP (2001). Pneumolysin is the main inducer of cytotoxicity to brain microvascular endothelial cells caused by *Streptococcus pneumoniae*. Infect Immun.

